# Lobetyolin, an anti-AD factor from the diet campanulaceae source, metabolism regulation and target exploration

**DOI:** 10.1007/s13659-025-00549-0

**Published:** 2025-09-12

**Authors:** Wen Huang, Yihan Liu, Haixin Jiang, Dongxue Guo, Yi Song, Junqi Wang, Luqi Li, Qiang Zhang

**Affiliations:** 1 Shaanxi Key Laboratory of Natural Products & Chemical Biology, College of Chemistry & Pharmacy, Northwest A&F University, Yangling, 712100 China; 2 Life Science Research Core Services, Northwest A&F University, Yangling, 712100 China

**Keywords:** Lobetyolin, AD prevention, Metabolic intervention, Glutathione metabolism, gst-38, gst-1

## Abstract

Bioactive compounds from food-compatible medicinal herbs have shown promise as preventive agents against age-related neurodegenerative conditions, particularly Alzheimer’s disease (AD). The present work aimed to find Lobetyolin as a new suppressor of Aβ aggregation and its interventions on abnormal metabolism in AD. Aβ-expressing *Caenorhabditis elegans* (strain CL4176) and wild-type worms were employed to evaluate paralysis onset, lifespan, cerebral Aβ deposition, and intracellular reactive oxygen species (ROS) after Lobetyolin administration. Untargeted ultra-high-performance liquid chromatography-high-resolution mass spectrometry (UHPLC-HRMS) metabolomics coupled with RNA-seq transcriptomics was carried out to profile systemic metabolic and gene-expression changes. Differential metabolites and transcripts were subjected to Kyoto Encyclopedia of Genes and Genomes (KEGG), Gene Ontology (GO), and pathway-impact analyses; hub targets were prioritized by integrating enrichment scores with in-silico docking. Lobetyolin (12.5–50 µM) markedly protected *C. elegans* from Aβ-driven toxicity and oxidative stress. In CL2006 worms, β-amyloid deposits fell by 54.8 ± 9.4%, while paralysis in CL4176 was delayed by 20.9 ± 4.5%. Lifespan increased by up to 18.2% in CL4176 and 25.0% in wild-type N2 worms. Concomitantly, intracellular ROS declined maximally by 28.1 ± 8.9% (N2) and 22.4 ± 3.8% (CL4176). Integrative metabolomic–transcriptomic analyses, validated by RT-qPCR, revealed selective remodeling of glutathione metabolism: gst-38 expression was suppressed, whereas gst-1 was elevated. Lobetyolin confers neuroprotective and geroprotective benefits in *vivo*, primarily through reprogramming glutathione-centered redox metabolism and selectively modulating glutathione-S-transferases (GST) isoforms. These findings position Lobetyolin as a promising dietary lead compound for AD prevention and healthy aging interventions.

**Graphical Abstract:**

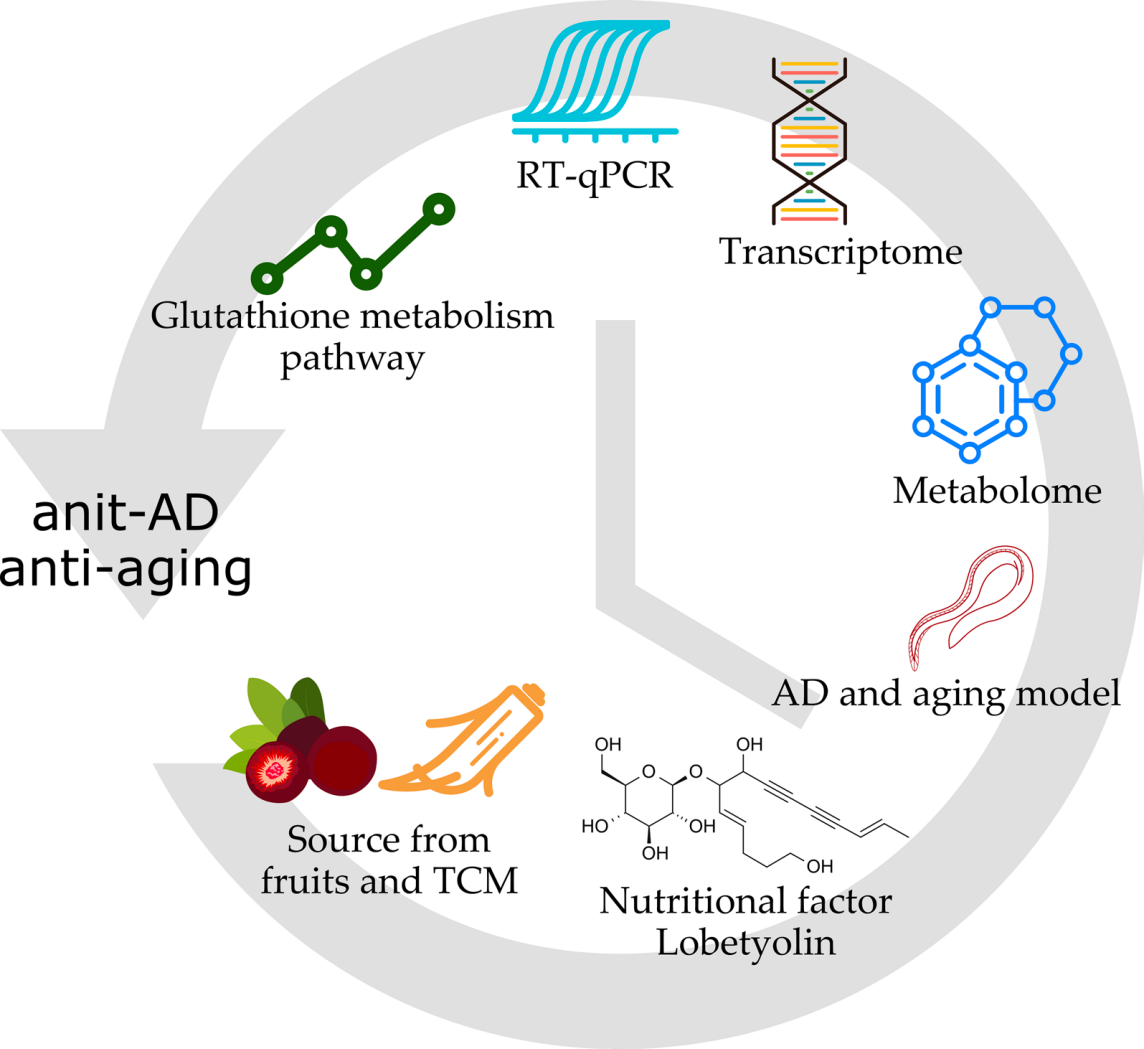

**Supplementary Information:**

The online version contains supplementary material available at 10.1007/s13659-025-00549-0.

## Introduction

Aging represents an irreversible and progressive decline in the functional integrity of cells, tissues, and organs, arising from the complex interplay of multifaceted factors [1]. This process is intricately linked to the escalating incidence of various age-related pathologies, including cancer, neurodegenerative disorders, cardiovascular diseases, and diabetes [2]. Consequently, contemporary anti-aging research has increasingly prioritized strategies to extend healthy lifespan and mitigate the deleterious impacts of senescence. Advancements in modern scientific methodologies have significantly deepened our understanding of aging mechanisms. For instance, the integration of metabolomics, genomics, transcriptomics, and microbiome analyses enables a holistic dissection of the molecular networks underpinning aging [3], thereby facilitating a more profound comprehension of its etiologies and accelerating the development of targeted anti-aging therapeutics.

Aging emerges as the predominant non-genetic contributor for Alzheimer’s disease (AD), with the senescent brain exhibiting numerous overlapping characteristics with the prodromal phases of AD. A hallmark pathological feature of AD is the accumulation of β-amyloid (Aβ), and aging is associated with elevated cerebral levels of Aβ_1–42_ [4]. Furthermore, Aβ_1–42_ functions as a mediator of oxidative stress, playing a pivotal role in AD pathogenesis [5] by diminishing superoxide dismutase (SOD) activity while elevating malondialdehyde (MDA) and reactive oxygen species (ROS) levels [6]. As a critical target in AD, Aβ is frequently employed in drug screening protocols and clinical diagnostics, underscoring its utility in therapeutic discovery and disease monitoring.

Transgenic *C. elegans strains* [7], characterized by their compact size, abbreviated lifespan, high reproductive fidelity, translucent anatomy, ease of cultivation, and the formation of muscle-associated Aβ deposits detectable by specific dyes—coupled with a progressive paralysis phenotype—have been extensively utilized to probe AD pathogenesis [8, 9]. Notable models such as CL4176 and CL2006 [10] facilitate in vivo screening of potential anti-AD compounds [11], offering a robust platform for mechanistic studies and pharmacological interventions. By leveraging these attributes, researchers can simulate age-related neurodegenerative processes, including the toxicity induced by Aβ, within a controlled and ethically sound environment. This approach not only bridges the gap between molecular insights and translational applications but also complements higher-order mammalian models, enhancing the efficiency of anti-aging and anti-AD drug discovery pipelines.

Chinese herbal medicines, often serving as dietary sources, are extensively utilized in anti-aging research [12, 13]. Our research group previously discovered that extracts from the fruit of *Cyclocodon lancifolius* prolonged the lifespan of *C. elegans* [14]. Subsequently, we isolated Lobetyolin, the characteristic anti-aging and anti-AD constituent, from the fruit of *C. lancifolius*. Lobetyolin is a distinctive polyacetylene glycoside associated with Platycodin. Herein, we report the newly discovered anti-aging capabilities of Lobetyolin and its protective effects against AD. Concurrently, we employed integrated metabolomics and transcriptomics approaches to elucidate the potential mechanisms of action and molecular targets for Lobetyolin.

## Results

### Lobetyolin reduced Aβ deposition in Aβ transgenic *C. elegans*

**Fig. 1:**
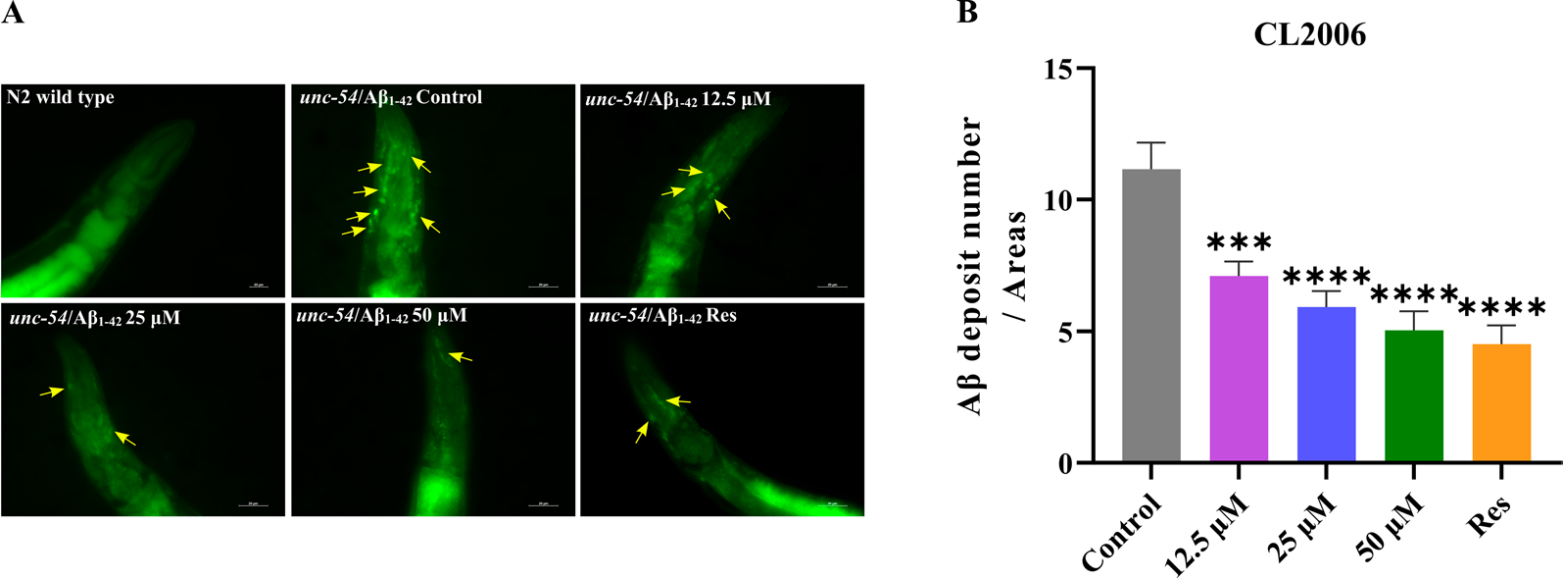
Lobetyolin reduced Aβ deposition in the transgenic *C. elegans* CL2006. **A** The ThT-stained Aβ deposits in transgenic *C. elegans* CL2006 and N2 strains served as genetic controls. **B** Quantification of Aβ_1–42_ deposits in each group was performed using ThT staining. (30 nematodes per group). Results are expressed as mean ± SEM, ***p < 0.001, ****p < 0.0001, compared to the control

The transgenic strain CL2006 continuously expresses human Aβ_1–42_ in body wall muscle cells, leading to excessive Aβ accumulation and progressive paralysis. [15]. To assess Aβ deposits, Thioflavin T (ThT) staining was employed for visualization and quantification. As shown in Fig. 1, ThT staining revealed prominent fluorescent plaques (yellow arrows) in the head region of CL2006 worms, indicative of Aβ aggregates, whereas no such deposits were observed in wild-type N2 controls. This confirmed the model’s specificity for Aβ pathology. Treatment with Lobetyolin at concentrations of 12.5, 25, and 50 μM significantly diminished the quantity of ThT-positive aggregates in the anterior region compared to untreated CL2006 controls (Fig. 1B, p < 0.05). Quantitative analysis demonstrated a dose-dependent reduction, with the 50 μM dose yielding the most pronounced effect (up to 54.8 ± 9.4% decrease in plaque count). Lobetyolin effectively reduces Aβ deposition in vivo, highlighting its potential as a therapeutic agent against AD by reducing proteotoxic stress.

### Lobetyolin postponed Aβ-induced paralysis in transgenic nematodes

**Fig. 2:**
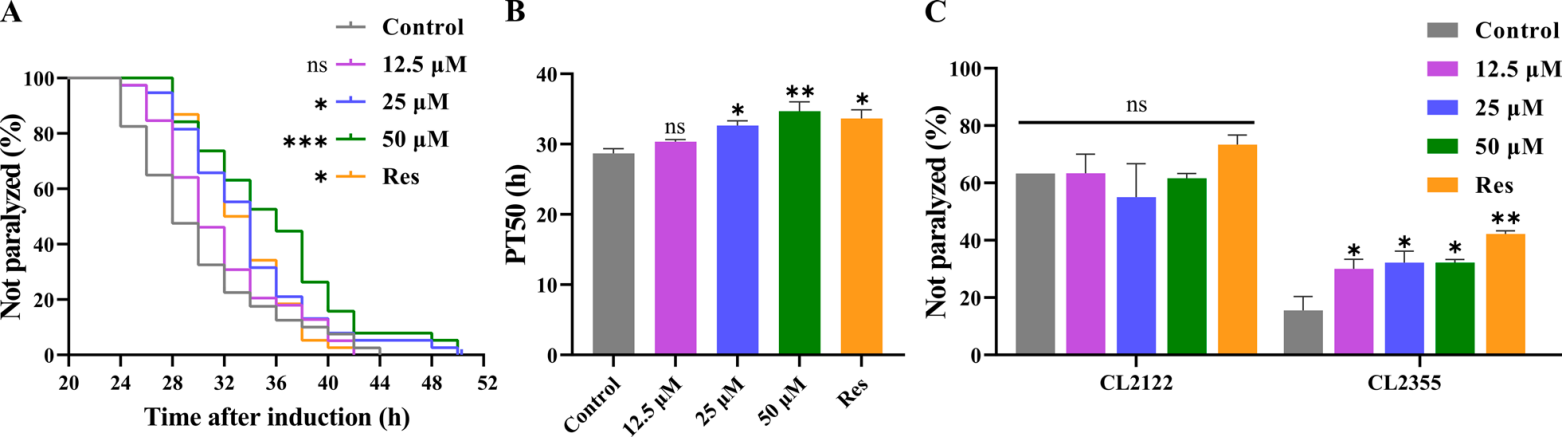
Lobetyolin delayed paralysis in transgenic *C. elegans* CL4176 induced by Aβ expression and improved the hypersensitivity of transgenic *C. elegans* CL2355 to exogenous 5-HT. **A** Curve of the unparalyzed rate (log-rank test); (**B**) Time to half paralysis (PT50) of *C. elegans*; (**C**) Impact of Lobetyolin on the hypersensitivity to 5-HT in the transgenic C. elegans strain CL2355, with CL2122 as the genetic control (30 nematodes per group, n = 4). Results are expressed as mean ± SEM; *p < 0.05, **p < 0.01, ***p < 0.001, compared to the control; ns, no significant difference

Since Lobetyolin was found to reduce Aβ deposition in transgenic nematodes, we further tested whether Lobetyolin could delay the paralysis of transgenic nematodes. At 25 °C, the CL4176 nematode accumulates Aβ_1-42_ deposits in its muscle cells., thereby causing paralysis symptoms and simulating some pathological characteristics of AD. The test results (Fig. 2A and B) showed that, compared with the control group, no significant delaying effect on Aβ-induced paralysis was detected in CL4176 nematodes fed from eggs with a low concentration of Lobetyolin at 12.5 µM. As the concentration of Lobetyolin was elevated, the CL4176 nematode showed a significant paralytic delay phenomenon, and this effect was positively correlated with the concentration of Lobetyolin (Fig. 2A). Further quantitative analysis was evaluated by calculating the PT_50_ value, which refers to the time required for half of the nematodes to show paralysis symptoms after paralysis was induced by an increase in environmental temperature. Compared with the control group (PT_50_ 28.67 ± 0.67 h), the PT_50_ of nematode groups treated with 25 μM and 50 μM Lobetyolin were significantly increased to 32.67 ± 0.67 h and 34.67 ± 1.33 h, respectively (Fig. 2B). These results support the potential of Lobetyolin in suppressing Aβ toxicity and also find that Lobetyolin can delay paralysis in transgenic nematodes.

### Lobetyolin alleviated the Aβ-induced 5-HT sensitivity in transgenic nematodes

Serotonin (5-HT) is an essential neurotransmitter in regulating behaviors such as olfaction, learning, oviposition, motility, and mating [16]. Exogenous 5-HT inhibits motility and causes paralysis in active nematodes. Neuronal Aβ overexpression renders the transgenic *C. elegans* strain CL2355 hypersensitive to exogenous 5-HT, thereby accelerating its paralysis phenotype [17]. The strain CL2122, which carries an identical genetic makeup but lacks the Aβ transgene, was used as the “no-Aβ” control to exclude any behavioral or fluorescence changes attributable to the transgenic backbone itself [18]. We thus examined 5-HT-induced paralysis in CL2355 nematodes under Lobetyolin treatment, with CL2122 nematodes as A genetic control, to confirm whether Lobetyolin could ameliorate neuronal dysfunction caused by Aβ protein. The results (Fig. 2C) revealed a notable reduction in the quantity of paralyzed CL2355 nematodes due to 5-HT after Lobetyolin treatment. In contrast, the control CL2122 nematode exhibited no statistically significant variations in the number of paralyzed worms under different treatment conditions. These results indicated that Lobetyolin effectively alleviated the 5-HT sensitivity induced by nematode Aβ, resulting in the effect of delaying paralysis.

### Lobetyolin prolonged the lifespan and reduced lipofuscin levels in *C. elegans*

**Fig. 3:**
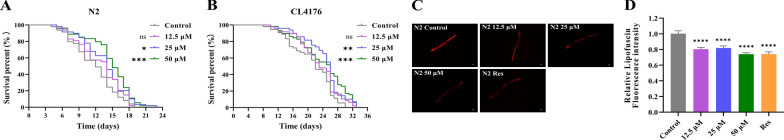
Survival curves of wild-type *C. elegans* strain N2 (**A**) and AD *C. elegans* CL4176 (**B**), fluorescence images (**C**), and quantitative analysis of lipofuscin fluorescence (**D**) in N2 *C. elegans* (30 nematodes per group). ns, no significant difference; *p < 0.05, **p < 0.01, ***p < 0.001, ****p < 0.0001, compared to the control

Aging represents the primary non-genetic risk factor for AD, with senescent brains exhibiting hallmarks akin to early AD pathology [4]. To explore the anti-aging potential of Lobetyolin, we assessed its impact on lifespan in wild-type and transgenic CL4176 (expressing human Aβ_1–42_) worms at concentrations of 12.5, 25, and 50 μM (Fig. 3A and B). In N2 worms, Lobetyolin at 25 and 50 μM significantly extended mean lifespan by 16.7–25% compared to DMSO controls (*p* < 0.01; Fig. 3A). Similarly, in the AD-like CL4176 model, these exposure concentrations (25 μM, 50 μM) prolonged lifespan by 18.2%, delaying age-related decline (*p* < 0.001; Fig. 3B). We further examined the effect of Lobetyolin on the accumulation of lipofuscin, an aging marker. The experimental data showed that the lipofuscin content in N2 nematodes treated with Lobetyolin (12.5, 25, and 50 μM) was markedly reduced relative to the untreated control (Fig. 3C and D). These findings substantiated the life-extending effect of Lobetyolin in wild-type worms and highlight its promise as an anti-aging compound.

### Lobetyolin reduced the reactive oxygen species (ROS) level

**Fig. 4:**
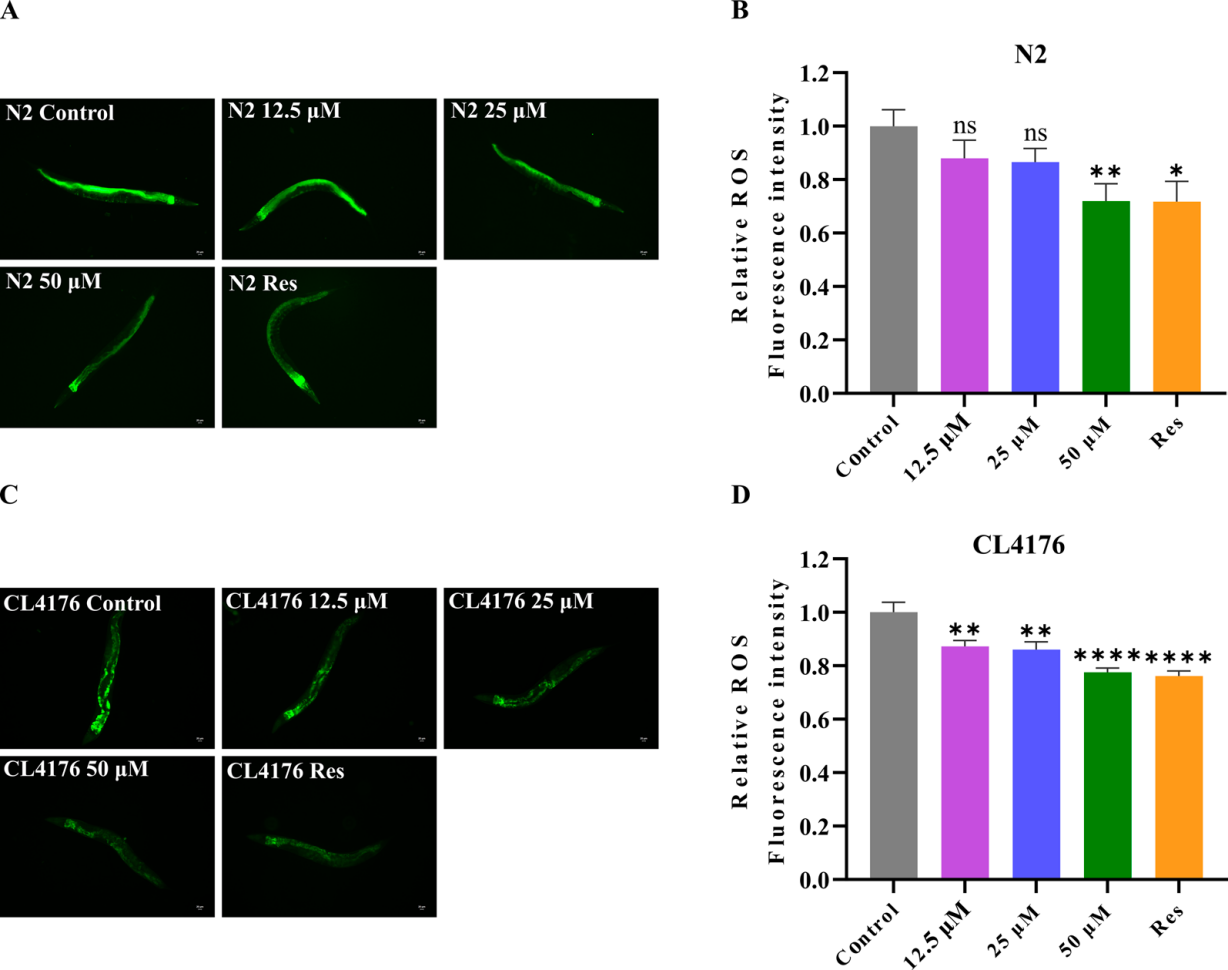
Lobetyolin reduced ROS in *C. elegans.* **A** Fluorescent photographs of wild-type *C. elegans* N2 to detect ROS with the indicator DCFH-DA; (**B**) Quantification of the mean fluorescence intensity in A (each group contained 30 *C. elegans*); (**C**) Fluorescent photographs of the AD *C. elegans* CL4176 to detect ROS with the indicator DCFH-DA; (**D**) Quantification of the average fluorescence intensity in C (each group contained 30 *C. elegans*). Results are expressed as mean ± SEM, ns, no significant difference; *p < 0.05, **p < 0.01, ****p < 0.0001, compared to the control

In line with the free radical theory of aging, excessive ROS is an important reason for the aging process [19]. It is reported that Lobetyolin has antioxidant effects [20]. To explore whether Lobetyolin delays the aging process of organisms through its antioxidant mechanism, especially the way of eliminating ROS in the body, in this study, DCFH-DA served as a free radical probe to evaluate the ROS levels in N2 nematodes treated with or without Lobetyolin. The results showed that at lower concentrations (12.5 and 25 μM), the inhibitory effect of Lobetyolin on ROS in N2 nematodes was relatively mild and did not show a significant reduction. However, when the concentration of Lobetyolin was increased to 50 μM, its antioxidant effect was significantly enhanced, effectively reducing the accumulation of ROS in N2 nematodes (Fig. 4A and B). This result indicates that the potential of Lobetyolin to extend lifespan is related to its ability to diminish the level of ROS in organisms.

Oxidative stress is also a crucial factor in the development of AD [21]. In A transgenic nematode model expressing human Aβ_1-42_, clear evidence of oxidative stress was observed, as evidenced by a marked elevation in superoxide levels prior to the development of paralytic symptoms. The first sign of oxidative stress is the massive accumulation of ROS. This accumulation not only impairs the normal function of the protease system but also promotes the aggregation of toxic proteins [22]. Conversely, mitochondrial dysfunction caused by proteotoxicity in turn exacerbates ROS overproduction [23], which in turn results in cognitive dysfunction in AD. To explore the antioxidant capacity of Lobetyolin against AD, we assessed the impact of Lobetyolin treatment on ROS levels in the transgenic nematode CL4176 using the DCFH-DA method. Lobetyolin lowered ROS generation in CL4176 nematodes in a dose-dependent manner: 12.5 μM cut ROS by 12.8 ± 3.8%, 25 μM by 14.0 ± 3.7%, and 50 μM by 22.4 ± 3.8% relative to untreated controls (Fig. 4C, D). These findings indicate that the postponed appearance of the Aβ-associated paralytic phenotype in CL4176 nematodes treated with Lobetyolin could be partly ascribed to the ROS-inhibiting effects of Lobetyolin.

### Safety assessment of Lobetyolin in *C. elegans*

**Fig. 5:**
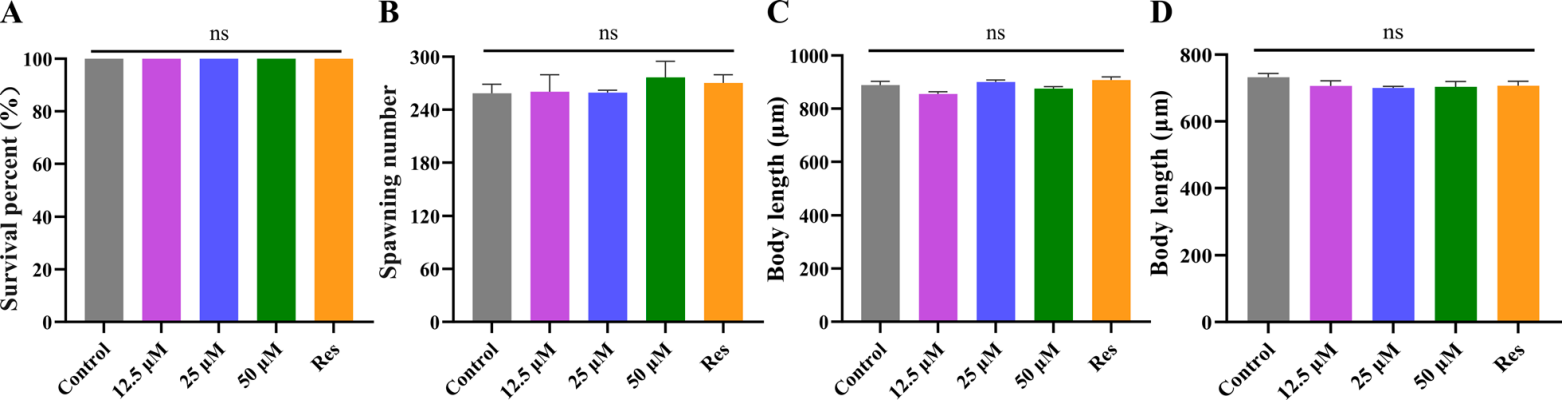
Lobetyolin did not influence the growth and development of *C. elegans.* **A** Acute toxicity of Lobetyolin to *C. elegans*; (**B**) The spawning numbers of *C. elegans*; (**C**) Wild-type *C. elegans* body length quantification; (**D**) Transgenic *C. elegans* CL4176 body length quantification (30 nematodes per group). Results are expressed as mean ± SEM; ns, no significant difference, compared to the control

To ensure safe dosing for subsequent assays, the acute toxicity of Lobetyolin was evaluated in L4-stage N2 worms. We evaluated how Lobetyolin influences *C. elegans* in terms of survival rate (Fig. 5A) and reproductive capacity (spawning number, Fig. 5B). Survival rates were monitored after 24 h exposure to Lobetyolin (12.5, 25, or 50 μM) or resveratrol (100 μM, positive control). No significant impact on viability was observed across all groups compared to DMSO controls Fig. 5A and B, (*p* > 0.05), confirming the safety of Lobetyolin at the tested concentrations.

To evaluate whether Lobetyolin treatment would interfere with the growth and development of nematodes, this study measured the body length of nematodes and the total number of their offspring. The findings revealed that, in comparison with the control group, after exposure to varying concentrations of Lobetyolin (12.5, 25, 50 μM) and resveratrol (100 μM) as the positive control, the body lengths of both N2 nematodes and CL4176 nematodes did not change significantly (Fig. 5C and D, Figure S1 in Supplementary Material). The above investigations indicate that Lobetyolin has no effect on the growth and development of nematodes.

### Metabolomics analysis of nematodes in the AD model by Lobetyolin

**Fig. 6:**
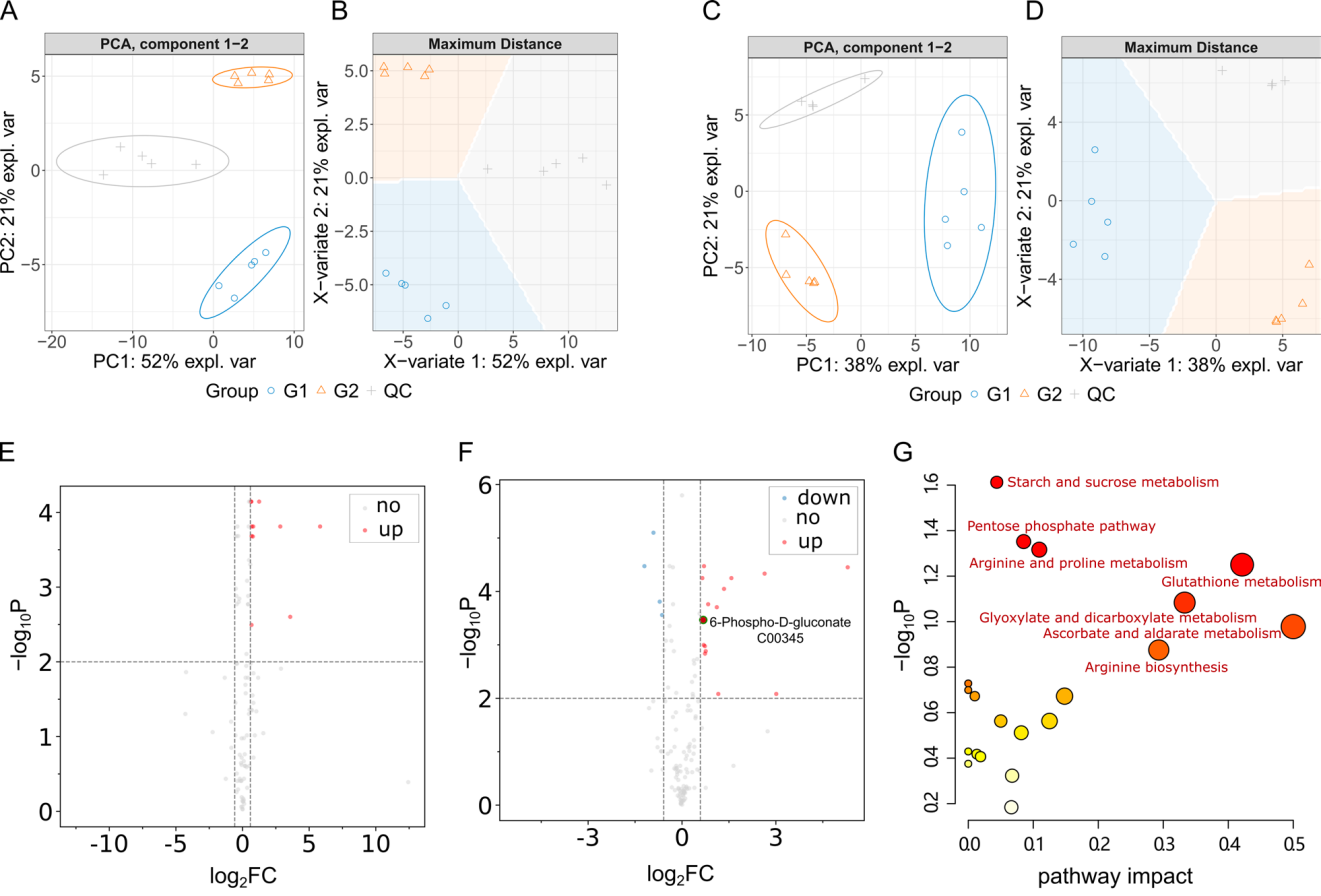
DMs and KEGG pathways associated with Lobetyolin treatment. **A**, **B** PCA and PLS-DA of metabolite profiles in the positive ion mode. **C**, **D** PCA and PLS-DA of metabolite profiles in the negative ion mode. **E** Volcano plot of differential metabolites in the positive ion mode. **F** Volcano plot of differential metabolites in the negative ion mode. **G** KEGG pathway enrichment analysis based on DMs. For detailed information on the differential metabolites, please see Table S1 in the Supplementary Materials

To unravel how lobetyolin suppressed Aβ aggregation and exerted its anti-AD effects, we implemented a combined transcriptomic–metabolomic strategy. Transcriptomics identified the upstream gene-level drivers, while metabolomics pinpointed the resulting metabolic alterations. The substantial alterations in the levels of differential metabolites observed between the blank group and the treated group suggest that the metabolic pathways have been profoundly impacted (Fig. 6A, B, C, D). This could be attributed to the ameliorative impact of Lobetyolin on nematode paralysis. We identified the key differential metabolites (DMs, FC > 2 and *p* < 0.02) that showed continuous (up-regulated or down-regulated) changes in the blank group and the administration group, as shown in Fig. 6E and F. To better understand the metabolite alterations induced by Lobetyolin, we conducted pathway enrichment analysis using the online toolkit Metaboanalyst 5.0 to explore and visualize the metabolic pathways affected by Lobetyolin. As shown in Fig. 6G, the pathway enrichment results of 20 metabolites with significant changes after Lobetyolin treatment of the CL4176 nematode. Among them, the *p*-value of starch and sucrose metabolism was the smallest, and the difference was the most significant. The influencing factors of ascorbate and aldarate metabolism and glutathione metabolism were relatively large.

**Fig. 7:**
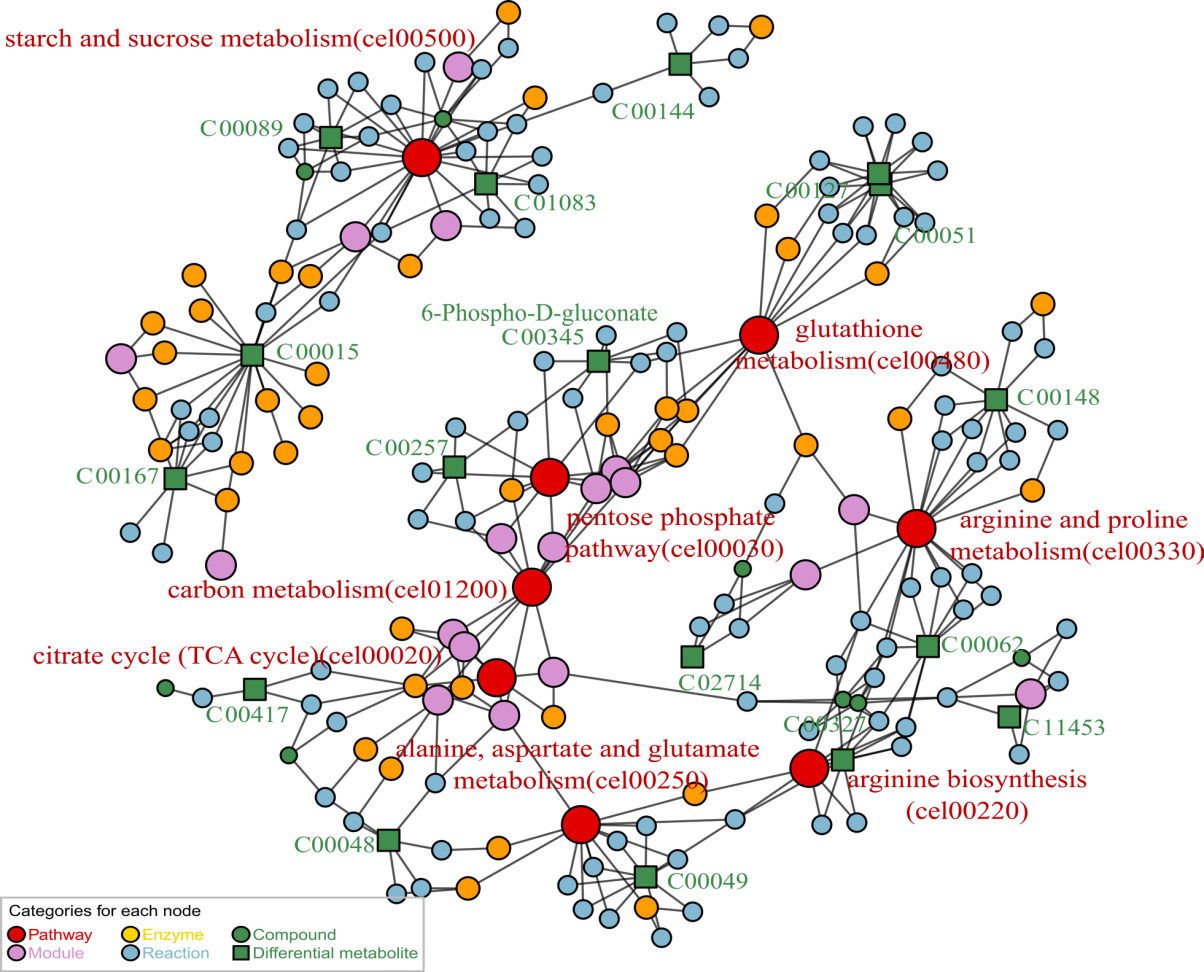
Metabolic pathway network constructed via FELLA enrichment. The numerical values represent the EC numbers of the enzymes. The nematodes were reared on NGM medium fortified with Lobetyolin (50 μM). The blank control group received no treatment. For enrichment analysis results, please refer to Table S2 in the Supplementary Materials

To more comprehensively depict the impacted pathways and their components, we constructed a map of metabolic pathway networks utilizing the FELLA package. The FELLA enrichment analysis identified seven metabolic pathways that match KEGG nodes (Fig. 7). Including pentose phosphate pathway (cel00030), arginine biosynthesis (cel00220), starch and sucrose metabolism (cel00500), and arginine and proline metabolism (cel00330), citrate cycle (TCA cycle) (cel00020), alanine aspartate and glutamate metabolism (cel00250), glutathione metabolism (cel00480). Combining the *p*-values from enrichment analysis, the influencing factors, and the hit count of DMs in pathways, we infer that the effects of Lobetyolin in alleviating paralysis and anti-aging are related to the pentose phosphate pathway (PPP). In addition, 6-Phospho-D-gluconate (C00345) (G6P) was found to be the differential metabolite related to PPP, and G6P was related to PPP and glutamate metabolism.

### Transcriptomic analysis of the AD model nematode by Lobetyolin

**Fig. 8:**
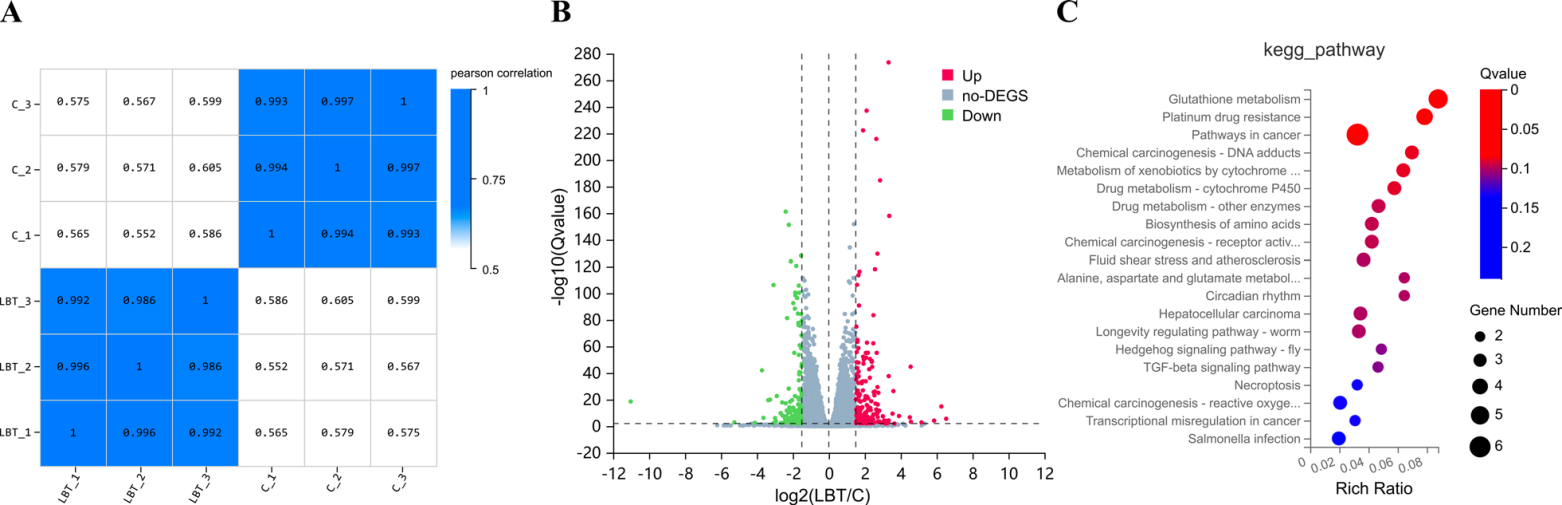
The results of enrichment analysis of DEGs in response to Lobetyolin. **A** Heat map of sample correlation; (**B**) Volcano plot of differential genes; (**C**) The 20 most significantly enriched pathways among DEGs in response to Lobetyolin, elucidated via KEGG pathway enrichment analysis. For enrichment analysis results, please refer to Tables S3 and S4 in the Supplementary Materials

Samples clustered tightly within each treatment group but showed low correlation across groups, indicating pronounced transcriptomic differences between the experimental conditions (Fig. 8A). The differential gene expression analysis of nematodes in each group is shown in Fig. 8B. Compared with the Control group, 323 differentially expressed genes (|log_2_FC|≥ 1.5, Qvalue ≤ 0.01) were identified in the treated group (Lobetyolin), including 185 upregulated genes and 138 downregulated genes. To further explore the role of Lobetyolin in alleviating paralysis, KEGG pathway enrichment (Q-value ≤ 0.05) was performed on these 323 genes (Fig. 8B). The top 20 enriched pathways associated with Lobetyolin relieving paralysis included glutathione metabolism, platinum drug resistance, and several others (Fig. 8C). Ranked by enrichment ratio and Q-value, the glutathione-metabolism pathway showed the strongest association. Consistently, metabolomic profiling pinpointed the same pathway as the central hub (Fig. 7) through which Lobetyolin modulates AD pathology, underscoring the agreement between the two analyses.

### RT-qPCR validation for the glutathione metabolism pathway

**Fig. 9:**
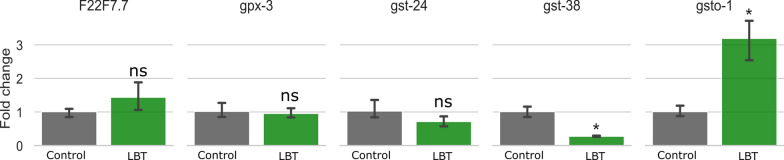
Effects of Lobetyolin on glutathione metabolism-related genes in *C. elegans* CL4176. Results are expressed as mean ± SEM, ns, no significant difference; *p < 0.05, compared to the control

According to the results of metabolomics and transcriptomics analysis, the Glutathione metabolism pathway was the most differentiated, so in the DEGs list, 5 genes (*F22F7.7*, *gpx-3*, *gsto-1*, *gst-38,* and *gst-24*) involved in the Glutathione metabolism pathway were selected for RT-qPCR validation. *Gsto-1* and *gst-38* showed significant differences (Fig. 9). The expression of *F22F7.7* and *gst-24* also increased and decreased to some extent. *Gsto-1* is a member of the GST family of genes, which plays an important role in cellular resistance to oxidative stress [24]. *Gst-38* is also a member of the GST family of genes, which is related to the antioxidant system [6]. The relationship between antioxidant potential and oxidative injury during the aging process has been observed in a wide range of tissues from diverse species. GST detoxifies a broad spectrum of endogenous and xenobiotic electrophilic toxins, including carcinogens, by catalyzing their conjugation with reduced glutathione (GSH). There is a positive correlation between GST activity and antioxidant capacity [25]. Therefore, the ratio of gene expression obtained by RT-qPCR proved the results of metabolomics and transcriptomics, and Lobetyolin can alleviate paralysis by regulating the expression of GST and participating in the process of glutathione metabolism, resulting in a certain anti-aging effect.

### Evaluation of Lobetyolin content in source plants

**Fig. 10:**
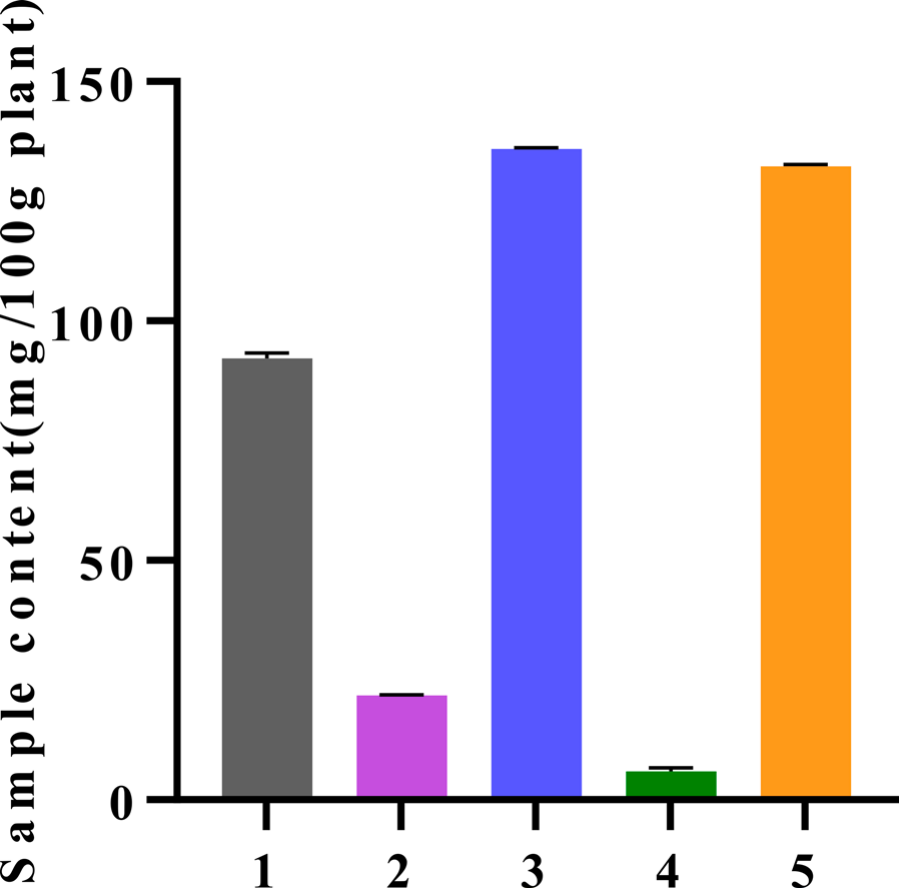
The content of Lobetyolin in different Chinese medicinal herbs. The calibration curve (R^2^ 0.9999) and the accompanying quantitative data are available in the Supporting Material (Fig. S2 and Table S5)

We further quantified Lobetyolin concentrations in a range of traditional Chinese medicines (TCMs), providing benchmark data for the development of TCM-based anti-aging products. Using HPLC, we quantified Lobetyolin levels in the radix of four medicinal species—*Codonopsis pilosula (Franch.) Nannf.* (1)*, Codonopsis tangshen Oliv.* (2)*, Codonopsis pilosula Nannf. var. modesta (Nannf.) L.T. Shen* (3)*, Adenophora tetraphylla (Thunb.) Fisch.* (4)*,* and a fruit from southern China (*Cyclocodon lancifolius*, 5)*.* The results are summarized in Fig. 10. Among them, the herbs 3 and 5 have higher content of Lobetyolin, 135.9 ± 0.1242 and 132.3 ± 0.2404 mg/100 g plant.

## Discussion

### First evidence of Lobetyolin’s inhibition of Aβ aggregation

Lobetyolin, a polyacetylene glycoside from *Codonopsis pilosula*, exhibits multi-targeted bioactivity. It suppresses gastric cancer cell proliferation by downregulating ASCT2-mediated glutamine uptake, modulating c-Myc, GSK3β, and AKT phosphorylation, and inducing ROS-mediated apoptosis via Nrf2 suppression, effectively inhibiting in vivo tumor growth [26]. In the nervous system, Lobetyolin shifts BV2 microglia from an M1 to M2 phenotype, decreases TNF-α and HIF-1α, increases TGF-β and CD206, and attenuates OGD/R injury through CKLF1/CCR4 signaling [27]. It also exerts antioxidant effects by lowering MDA and restoring glutathione levels [28].

To our knowledge, this research represents the first demonstration that Lobetyolin markedly inhibits Aβ aggregation. This bioactivity not only alleviates Aβ-induced paralysis in AD models but also confers significant anti-aging benefits, including extended lifespan and enhanced stress resistance. Previous in vitro reports suggest interaction with BACE1 [29]; our in vivo data in *C. elegans* are consistent with reduced Aβ burden but do not establish enzyme-level causality. These findings supported BACE1 as a plausible molecular target for Lobetyolin to inhibit amyloidogenic action and provided a basis for subsequent rodent studies and early translational exploration. However, Evidence for Lobetyolin’s direct interaction with Aβ and its corrective impact on AD metabolic dysregulation remains scarce, hindering its rational development as a neuroprotective food factor or drug lead. Therefore, an integrated study linking Lobetyolin exposure with AD-specific metabolic perturbations and the molecular events of Aβ production, aggregation, and clearance is essential to elucidate their intrinsic relationship and to justify dietary or therapeutic use against age-related cognitive decline.

Inspired by the observed neuroprotection effects, the aglycone, Lobetyol, and related ginsenoyne-type polyacetylenes merit consideration as comparators. Reports of Lobetyol’s antioxidant activity [30] suggest potential convergence on Aβ aggregation and redox-linked longevity phenotypes, although this remains untested in our study. Notably, aglycones may possess physicochemical properties (e.g., distribution coefficient, logD at pH 7.4) that favor blood–brain barrier penetration and target engagement relative to glycosides. Rigorous head-to-head evaluation—encompassing in vitro aggregation assays, *C. elegans* phenotypes, and rodent pharmacokinetics/brain exposure—will be necessary to determine whether deglycosylation enhances central efficacy and to prioritize candidates for subsequent preclinical development.

### Glutathione metabolism as the principal intervention pathway underlying Lobetyolin’s anti-AD activity

Our integrated metabolomic and transcriptomic analyses, corroborated by RT-qPCR validation and phenotypic observations, demonstrated that Lobetyolin’s inhibition of Aβ aggregation prioritized by integrated omics as a central pathway; RT‑qPCR showed modulation of GST-related transcripts (e.g., *gsto-1*, *gst-38*), supporting the hypothesis that redox homeostasis is involved in the observed phenotypes.

Glutathione metabolism and Aβ pathology reinforce each other: a lowered GSH/GSSG ratio amplifies oxidative stress, while Aβ oligomers elevate ROS that further depletes glutathione, sustaining a vicious cycle [31]. Pyruvate can modulate glutathione peroxidase/reductase activities, and PI3K/AKT/Glut1 activation improves glucose metabolism and elevates glutathione, countering Aβ damage [32]. Moreover, impaired glutathione turnover exacerbates NF-κB–mediated neuroinflammation and Aβ deposition, whereas glutathione augmentation suppresses microglial overactivation and promotes Aβ clearance via GPR55 signaling [33]. Clinically, plasma glutathione inversely correlates with Aβ PET positivity, indicating early redox imbalance [31]. In our study, transcriptomic enrichment prioritized glutathione-centered redox metabolism, concordant with reduced ROS, mitigated Aβ‑linked paralysis, and lifespan extension in *C. elegans*. Together, these data support glutathione homeostasis as a pathway-level target and a foundation for subsequent mammalian testing.

### Lobetyolin: a dual food-medicine nutraceutical for AD prevention

Lobetyolin, a C14‐polyacetylene glycoside from *Codonopsis pilosula*, embodies the food–medicine duality of traditional radix *Codonopsis*, with established safety in dietary and herbal use [34]. Our analysis showed that Lobetyolin occurs at the highest levels—above 0.1% (w/w)—in just two materials: the fruit of *Cyclocodon lancifolius* and the root of *Codonopsis pilosula* var. modesta, the latter being commercially known as *Codonopsis* radix. Taken together with their established food-medicine use, these data highlight Lobetyolin as a potent nutritional factor that could be incorporated into the diet to help prevent AD and slow aging.

### Limitations and future directions

Although the *C. elegans* data coherently implicate glutathione-centered redox metabolism in Lobetyolin’s effects across multiple phenotypes (Aβ burden, paralysis, ROS, and lifespan), important gaps constrain mechanistic certainty and translational inference. Chief among these is the absence of mammalian validation, as no rodent behavioral, biochemical, or histopathological evidence currently corroborates the nematode findings. Equally, pharmacokinetic properties—including absorption, distribution, metabolism, brain exposure, and clearance—remain undefined, limiting dose extrapolation, target engagement assessment, and the design of exposure-matched efficacy studies in higher organisms. Safety considerations also require systematic evaluation beyond acute nematode assays; comprehensive toxicology encompassing systemic and organ-specific toxicity, genotoxicity, and chronic safety in mammals has not yet been undertaken.

The present work does not advance clinical claims. Rather, these findings are positioned as a mechanistic foundation to guide subsequent rodent studies and early translational development. Priorities include establishing mammalian efficacy in disease-relevant models, elucidating PK/PD relationships with confirmed brain penetration, and conducting tiered safety profiling. By delineating these needs, the study provides a clear roadmap for advancing Lobetyolin from pathway prioritization toward rigorous preclinical validation.

## Materials and methods

### Materials

Lobetyolin was isolated from the fruits of *C. lancifolius*. Briefly, dried fruits were pulverized and subjected to ethanol extraction, followed by fractionation using column chromatography (silica gel and reversed-phase HPLC) to yield purified Lobetyolin. Additionally, high-purity Lobetyolin (≥ 98% as determined by HPLC) was commercially sourced from Shanghai Yingxin Laboratory Equipment Co., Ltd. (Shanghai, China). The chemical structure of both isolated and purchased Lobetyolin was verified through spectroscopic analyses, including ^1^H and ^13^C NMR (Fig. S3 in the Supplementary Material) [35]. β-Mercaptoethanol and Tris-base were sourced from Aladdin Biochemical Technology Co., LTD. (Shanghai, China). Riboflavin T (ThT), paraformaldehyde, Triton X-100, and 5-hydroxytryptamine (5-HT) were sourced from McLean Biochemical Technology Co., LTD. (Shanghai, China). The dimethyl sulfoxide (DMSO) was acquired from Solarbio Life Sciences Co., LTD. (Beijing, China).

### *C.elegans* strain and maintenance conditions

All *C. elegans* strains (wild-type N2; CL4176, dvIs27 [*myo-3p::*Aβ_1–42_*::let-851 3′utr)* + *rol-6 (su1006)*]; CL2006, dvIs2 [*pCL12 (unc-54/*human Aβ_1–42_ minigene*)* + *ROre-6 (su1006)*]; CL2122, dvIs15 [*(pPD30.38) unc-54 (vector)* + *(pCL26) mtl-2::GFP*]; CL2355, dvIs50 [*pCL45 (snb-1::*Aβ_1–42_*: 3′utr (long)* + *mtl-2::GFP]*) were sourced from the Caenorhabditis Genetics Center (CGC) at the University of Minnesota, MN, USA. The uracil-auxotrophic *Escherichia coli* OP50 strain, used as a food source for the nematodes, was purchased from Shangyuan Bioscience (Fujian, China). Supplementary Table S6 offers a complete overview of the *C. elegans* strains utilized in this study.

### Toxicity evaluation in *C.elegans*

Acute toxicity assays were conducted on synchronized L4-stage N2 *C. elegans* [36]. Lobetyolin and resveratrol (positive control) were diluted in K medium (32 mM KCl, 51 mM NaCl) to final concentrations of 12.5, 25, and 50 μM for Lobetyolin, and 100 μM for resveratrol. In 96-well plates, 200 μL of each test solution was added per well, followed by 30 worms. The control group received K medium containing 0.1% DMSO. The plates were maintained at 20 °C for a duration of 24 h, following which the quantity of deceased nematodes in each group was documented via microscopic observation.

### Body length determination

We followed a previously reported protocol with minor modifications [14]. Briefly, synchronized L4-stage N2 and CL4176 *C. elegans* were transferred to nematode growth medium (NGM) plates supplemented with control (0.1% DMSO), Lobetyolin (12.5, 25, or 50 μM), or positive control (100 μM resveratrol). N2 worms were cultured at 20 °C for 4 days, whereas CL4176 worms were kept at 15 °C for 6 days. Body lengths were imaged utilizing an inverted fluorescence microscope (DMi8, Leica, Germany). Image analysis was performed using ImageJ 1.54f (National Institutes of Health, USA) to quantify worm body lengths.

### Reproductive evaluation in *C.elegans*

The synchronized L4-stage wild-type nematodes were relocated to a new NGM medium containing the control (0.1% DMSO), Lobetyolin (12.5, 25, and 50 μM), and positive control (100 μM resveratrol), a single worm being placed on each plate. To distinguish the offspring from the parent worms, the worms were transferred to fresh plates daily until the end of the reproductive period. The plates were kept at 20 °C, and the number of offspring was counted once they reached the L3 stage.

### Life span determination of *C.elegans*

The synchronized L4 N2/CL4176 worms were transferred to NGM plates containing control (0.1% DMSO), Lobetyolin (12.5, 25, and 50 μM), and positive control (100 μM resveratrol). The N2 and CL4176 worms were respectively cultivated at 20 °C and 15 °C with at least 60 nematodes per plate. NGM plates were replaced every two days during incubation until all worms had perished. Daily observations recorded the number of normally deceased worms. Death was confirmed by the absence of response to gentle stimulation with a platinum wire. Worms that crawled to the plate edge and desiccated were considered abnormal deaths.

### Determination of lipofuscin in *C.elegans*

Synchronized L4-stage N2 worms were relocated to NGM plates supplemented with control (0.1% DMSO), Lobetyolin (12.5, 25, or 50 μM), or resveratrol (100 μM, positive control) and cultured for 10 days at 20 °C. Worms were collected, anesthetized with 5 mM levamisole, and mounted on glass slides. Individual worms were imaged utilizing a DMi8 inverted fluorescence microscope (Leica, Germany) at 530 nm excitation. Lipofuscin autofluorescence was captured for ≥ 30 worms per group, with fluorescence intensity quantified via ImageJ software.

### Paralysis rate assay in Aβ-transgenic CL4176

The transgenic strain CL4176 expresses human Aβ_1–42_ upon temperature upshift from 15 °C to 25 °C, inducing an AD-like paralysis phenotype while maintaining normal growth at 15 °C. This model was employed to evaluate the protective effects against paralysis for Lobetyolin. More than 40 synchronized CL4176 eggs per group were transferred to NGM plates supplemented with control (0.1% DMSO), Lobetyolin (12.5, 25, or 50 μM), or resveratrol (100 μM, positive control). The plates were initially incubated at 15 °C for 48 h and then shifted to 25 °C for an additional 24 h to trigger Aβ expression. Subsequently, the non-paralysis rates were assessed every 2 h. Paralysis was determined as the condition where the worms exhibited only head movement and no body response to repeated stimulation with a platinum wire [11].

### Assessment of serotonin hypersensitivity in transgenic *C. elegans*

The CL2355 strain expresses human Aβ_1–42_ in neurons, leading to a paralytic response to exogenous serotonin (5-hydroxytryptamine, 5-HT). In contrast, the CL2122 strain, which lacks neuronal Aβ_1–42_ expression, serves as a control strain that does not respond to serotonin stimulation [37]. Synchronized eggs from the CL2355 and CL2122 strains were relocated to NGM plates supplemented with control (0.1% DMSO), Lobetyolin (12.5, 25, or 50 μM), or resveratrol (100 μM, positive control). The plates were initially incubated at 15 °C for 3.5 days and then shifted to 25 °C for 1.5 days. Subsequently, 30 worms from each group were exposed to a 5 mg/mL solution of 5-HT for 5 min, and the paralysis rates were assessed based on the lack of movement.

### Quantification of Aβ deposits in *C. elegans*

Aβ deposition in worms was assessed using Thioflavin T (ThT) staining. Synchronized CL2006 eggs were transferred to NGM plates supplemented with control (0.1% DMSO), Lobetyolin (12.5, 25, or 50 μM), or resveratrol (100 μM, positive control) and incubated at 20 °C until day 8 of adulthood. The worms were harvested, washed with M9 buffer, and subsequently fixed in a 4% paraformaldehyde solution at 4 °C for 24 h. After performing three washes with M9 buffer, permeabilization was carried out using an infiltration solution composed of 1% Triton X-100, 5% β-mercaptoethanol, and 125 mM Tris–HCl (pH 7.4) at 37 °C for 24 h. The worms were then washed three times with M9 buffer and stained with 0.125% Thioflavin T (ThT) for 2 min. Background fluorescence was eliminated through repeated washes with 50% ethanol. Finally, the worms were mounted onto slides and imaged using an inverted fluorescence microscope (DMi8, Leica, Germany). Aβ plaques were quantified by counting ThT-reactive deposits in the anterior region (head) of 30 worms per group.

### Quantification of ROS in *C. elegans*

Intracellular reactive ROS levels were assessed using the fluorescent probe 2′,7′-dichlorofluorescein diacetate (DCFH-DA). Synchronized N2 and CL4176 eggs were plated on NGM with control (0.1% DMSO), Lobetyolin (12.5, 25, or 50 μM), or resveratrol (100 μM) and incubated at 20 °C (N2) or 15 °C (CL4176). Adult worms (day 8 N2; day 10 CL4176) were placed in 96-well plates with M9 buffer containing 50 μM DCFH-DA, then incubated dark for 2 h at respective temperatures. After M9 washes, worms were anesthetized (5 mM levamisole) on slides and imaged via DMi8 microscope (Leica, Germany) at 470 nm excitation, with 30 worms per group. Fluorescence intensity was quantified with ImageJ.

### Sample preparation for LC/MS metabolomics analysis

Metabolomics samples were divided into G1 (CL4176 with 0.1% DMSO, AD model) and G2 (CL4176 with 50 μM Lobetyolin), with 5 replicates each. Synchronized eggs were seeded onto appropriate NGM plates, cultured at 15 °C for 48 h, and then moved to 25 °C to trigger Aβ expression. Worms were harvested at 26 h post-induction (G2 at 90% non-paralysis time), washed with M9 buffer and distilled water, rapidly frozen in liquid Nitrogen, and subsequently freeze-dried. Dry weights were recorded. Metabolites were extracted using 80% methanol with 10 μM 2-bromo-L-phenylalanine (internal standard) at a 1:200 (mg: μL) ratio. Samples were homogenized on ice, centrifuged at 12,000 rpm for 10 min at 4 °C, and supernatants collected. QC samples were prepared by pooling 10 μL from each. All were stored at − 20 °C before testing.

### LC–MS/MS analysis and data processing

Samples and QC samples were analyzed via LC–MS/MS on a Q Exactive Focus Orbitrap system (Thermo Fisher Scientific, USA) [38]. Briefly, 5 μL samples were separated on a Hypersil Gold™ aQ C18 column (150 × 2.1 mm, 1.9 μm) at 30 °C, eluting with a gradient of MeCN (containing 0.1% AcOH) at 0.3 mL/min. MS scans in positive/negative modes used ddMS^2^ (m/z 70–1000), with MS1/MS2 resolutions of 70,000/35,000 and stepped collisions (10, 20, 40 V).

All supernatants and QC samples were injected into LC–MS/MS for analysis with analytical parameters set according to previously reported methods [38]. In summary, LC–MS/MS analysis was conducted using a Q Exactive Focus Orbitrap LC–MS/MS system (Thermo Fisher Scientific, Waltham, MA, USA). The 5.0 μL sample was separated on an aQ C18 Polar column of Hypersil Gold^™^ (1.9 μm, 150 × 2.1 mm) maintained at 30 °C. The mobile phase consisted of H_2_O (A)-acetonitrile (B), both of which contained 0.1% (v/v) acetic acid. The column was eluted with a gradient (1–100% in 15 min) of Mobile Phase B at a flow rate of 0.3 mL/min. Mass spectra were scanned in positive and negative ion modes with step collisions (10, 20, and 40 V) using the ddMS^2^ scheme. Full MS and MS/MS scans ranged from 70 to 1000 m/z, with MS1 solution 70000 and MS2 35000.

Data analysis and visualization were performed with reference to a previous study from our laboratory, with minor modifications [39]. The mixOmics package in Bioconductor R software was utilized for PCA and OPLS-DA analyses. Statistical p-values were determined using a t-test in R software. The thresholds of FC > 1.5 2 and p < 0.02 were used to screen differential metabolites. Volcano diagrams were generated using the R software package. Finally, using online tools MetaboAnalyst 5.0 [40] and FELLA package (version 1.14) [41] to determine the differences in metabolite pathways of enrichment and analysis.

### Transcriptomics analyses of transgenic CL4176 C. elegans

Sample preparation and collection methods for transcriptomics were the same as those for metabolomics, as in 4.12. The sample data were analyzed in the BGI system on https://biosys.bgi.com (accessed on May 15, 2025) for genetic screening of differentially expressed genes (DEGs) and enrichment.

### RT-qPCR analysis in transgenic CL4176

The collected samples were processed identically to those in the metabolomics analysis, as detailed in Sect. 4.12, and were divided into two groups: the blank controls and the Lobetyolin treatment group, each with 3 replicates. Primer design was carried out by Shenggong Biotechnology Co., Ltd. (Shanghai, China). Total RNA extraction was performed using the AG RNAex Pro reagent (Accurate Biology, Changsha, China) following the manufacturer's protocol. RT-qPCR was performed on a CFX 96 Touch instrument (Bio-Rad Laboratories, Inc., Hercules, CA, USA). The relative expression levels were determined using the 2^−∆∆Ct^ and normalized to reference genes.

### Determination of Lobetyolin content in different Chinese medicinal materials

The standard curve was drawn, and the standard Lobetyolin (98%, Shanghai Yingxin Laboratory Equipment Co., Ltd. Shanghai, China) was accurately prepared into 0.5 mg/ml, 0.25 mg/ml, 0.1 mg/ml, 0.05 mg/ml and 0.01 mg/ml standard solutions with methanol, and the detection wavelength was set at 210 nm. Then the chromatographic conditions were screened (equal elution with 47% methanol), and the peak area was measured by Agilent 1100 HPLC (Agilent Technologies Co., Ltd, Santa Clara, CA, USA) as the ordinate, and the standard curve was drawn according to the actual mass concentration of Lobetyolin. After that, the sample solution was prepared and added with 100% methanol at a ratio of 1:5 (g: ml), sonicated for 40 min, and then mixed after cooling. The supernatant was taken and filtered to obtain the sample solution. The peak area was measured and substituted into the standard curve to calculate the content of codonopsis side in Chinese herbal medicine.

### Data analysis

All the data were analyzed with R packages and visualized using ggplot2. One-way ANOVA was performed and followed by Dunnett's multiple comparisons test or log-rank test. Data are presented as mean ± SEM. Asterisks (*) indicate significant differences (ns, not significant; *p < 0.05, ** *p* < 0.01, *** *p* < 0.001, **** *p* < 0.0001).

## Supplementary Information

Supplementary Material 1.

## Data Availability

All data generated or analyzed during this study are included in this published article. The full data of transcriptome and metabolome are available from the corresponding author on reasonable request.
