# Supplementary material for: Lobetyolin, an anti-AD factor from the diet campanulaceae source, metabolism regulation and target exploration
Source: Nat Prod Bioprospect. 2025 Sep 12;15(1):64. doi: 10.1007/s13659-025-00549-0 (PMC12431999; doi:10.1007/s13659-025-00549-0)
Supplement: Supplementary file 1 — Supplementary Material 1. [file 13659_2025_549_MOESM1_ESM.docx]

***Supporting Information***

**Lobetyolin, An Anti-AD Factor From the Diet Campanulaceae Source, Metabolism Regulation And Target Exploration**

Wen Huang^1,†^, Yihan Liu ^1,†^, Haixin Jiang^1^, Dongxue Guo^1^, Yi Song^1^, Junqi Wang^1^, Luqi Li^2^, Qiang Zhang*^,1^

1. Shaanxi Key Laboratory of Natural Products & Chemical Biology, College of Chemistry & Pharmacy, Northwest A&F University, Yangling 712100, China
2. Life Science Research Core Services, Northwest A&F University, Yangling 712100, China

† These authors contributed equally to this work.

***** Correspondence: [zhangq@nwsuaf.edu.cn](mailto:zhangq@nwsuaf.edu.cn) (Q. Z.)

**Figure S1.** Lobetyolin had no effect on the growth and development of *C. elegans.* (A) Wild-type *C. elegans* images and (B) transgenic *C. elegans* CL4176 images.


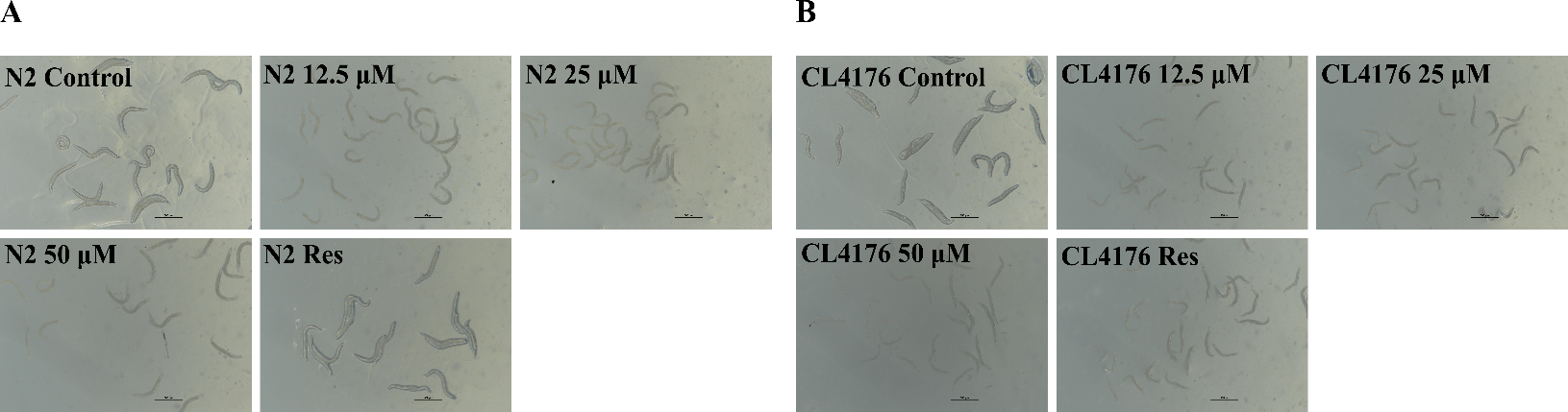


# **Table S1.** Differential metabolites between Lobetyolin-treated and control group

|  | KEGG ID | Metabolite name | Average Rt(min) | Average Mz | Adduct type | Formula | ion mode | log_2_FC | −lg(fdr) | regulation |
| --- | --- | --- | --- | --- | --- | --- | --- | --- | --- | --- |
| 1 | C00051 | L-Glutathione (reduced) | 1.391 | 308.0903 | [M+H]^+^ | C_10_H_17_N_3_O_6_S | positive | 0.66 | 4.14 | up |
| 2 | C00583 | Propylene glycol | 1.277 | 147.1126 | [M+ACN+NH_4_]^+^ | C_3_H_8_O_2_ | positive | 0.77 | 3.68 | up |
| 3 | C00144 | Guanosine monophosphate | 1.822 | 364.0642 | [M+H]^+^ | C_10_H_14_N_5_O_8_P | positive | 0.63 | 4.14 | up |
| 4 | C12136 | Epigallocatechin | 2.364 | 307.0827 | [M+H]^+^ | C_15_H_14_O_7_ | positive | 0.67 | 2.49 | up |
| 5 | C00049 | Aspartic acid | 1.210 | 134.0445 | [M+H]^+^ | C_4_H_7_NO_4_ | positive | 1.25 | 4.14 | up |
| 6 | C00148 | Proline | 1.372 | 116.0706 | [M+H]^+^ | C_5_H_9_NO_2_ | positive | 0.68 | 3.68 | up |
| 7 | C00327 | L-Citrulline | 1.315 | 176.1026 | [M+H]^+^ | C_6_H_13_N_3_O_3_ | positive | 5.82 | 3.81 | up |
| 8 | C02714 | N-acetyl putrescine | 1.931 | 131.1178 | [M+H]^+^ | C_6_H_14_N_2_O | positive | 0.80 | 3.81 | up |
| 9 | C07572 | Citalopram | 5.029 | 155.9739 | [M–C_14_H_23_+Na]^+^ | C_20_H_21_FN_2_O | positive | 3.58 | 2.60 | up |
| 10 | C00021 | S-Adenosyl-L-homocysteine | 5.381 | 385.1281 | [M+H]^+^ | C_14_H_20_N_6_O_5_S | positive | 0.69 | 3.81 | up |
| 11 | C01760 | Vomifoliol | 16.701 | 279.1582 | [M+MeOH+Na]^+^ | C_13_H_20_O_3_ | positive | 2.83 | 3.81 | up |
| 12 | C00089 | Sucrose | 1.323 | 401.1303 | [M+CH_3_COO]^−^ | C_12_H_22_O_11_ | negative | -0.91 | 5.10 | dn |
| 13 | C00257 | Gluconic acid | 1.232 | 195.0504 | [M−H]^−^ | C_6_H_12_O_7_ | negative | 0.69 | 3.00 | up |
| 14 | C00144 | Guanosine monophosphate | 1.817 | 362.0510 | [M−H]^−^ | C_10_H_14_N_5_O_8_P | negative | 0.65 | 4.25 | up |
| 15 | C00049 | L-aspartate | 1.228 | 132.0291 | [M−H]^−^ | C4H7NO4 | negative | 0.84 | 3.76 | up |
| 16 | C09822 | Cyclohexane-1-carboxylate | 16.743 | 301.1663 | [2M+HCOO]^−^ | C_7_H_12_O_2_ | negative | -0.64 | 3.56 | dn |
| 17 | C01083 | Trehalose | 1.323 | 341.1090 | [M−H]^−^ | C_12_H_22_O_11_ | negative | -0.71 | 3.81 | dn |
| 18 | C00327 | Citrulline | 1.306 | 174.0876 | [M−H]^−^ | C_6_H_13_N_3_O_3_ | negative | 5.29 | 4.45 | up |
| 19 | C00062 | L-arginine | 1.314 | 131.0815 | [M−CH_3_N_2_]^−^ | C_6_H_14_N_4_O_2_ | negative | 2.64 | 4.33 | up |
| 20 | C00127 | L-Glutathione oxidized | 2.368 | 611.1456 | [M−H]^−^ | C_20_H_32_N_6_O_12_S_2_ | negative | 0.76 | 2.88 | up |
| 21 | C00167 | Uridine diphosphate glucuronic acid | 1.090 | 579.0275 | [M−H]^−^ | C_15_H_22_N_2_O_18_P_2_ | negative | 1.58 | 4.25 | up |
| 22 | C15971 | Dihydrophaseic acid | 14.351 | 239.1288 | [M−C_2_H_3_O]^−^ | C_15_H_22_O_5_ | negative | 0.73 | 2.98 | up |
| 23 | C00345 | 6-phosphogluconate | 1.094 | 275.0176 | [M−H]^−^ | C_6_H_13_O_10_P | negative | 0.63 | 3.50 | up |
| 24 | C03947 | Glycerol 1,2-cyclic phosphate | 1.176 | 152.9949 | [M−H]^−^ | C_3_H_7_O_5_P | negative | 0.74 | 2.84 | up |
| 25 | C00015 | Uridine 5'-diphosphate | 1.102 | 402.9950 | [M−H]^−^ | C_9_H_14_N_2_O_12_P_2_ | negative | 1.11 | 3.70 | up |
| 26 | C00021 | S-Adenosyl-homocysteine | 5.392 | 383.1147 | [M−H]^−^ | C_14_H_20_N_6_O_5_S | negative | 0.70 | 4.47 | up |
| 27 | C11453 | 2-C-Methyl-D-erythritol | 1.060 | 276.9884 | [M−H]^−^ | C_5_H_12_O_9_P_2_ | negative | 1.34 | 4.05 | up |
| 28 | C00108 | Anthranilic acid | 9.426 | 136.0394 | [M−H]^−^ | C_7_H_7_NO_2_ | negative | -1.20 | 4.47 | dn |
| 29 | C00048 | Glyoxylate | 2.025 | 72.9919 | [M−H]^−^ | C_2_H_2_O_3_ | negative | 1.16 | 2.08 | up |
| 30 | C00417 | Cis-Aconitic acid | 18.904 | 103.0026 | [M−C_3_H_3_O_2_]^−^ | C_6_H_6_O_6_ | negative | 3.01 | 2.08 | up |

* fdr, FDR-adjusted *p*-value

# **Table S2.** Results of FELLA enrichment analysis.

| KEGG id | Type | KEGG.name | −lg(p value) |
| --- | --- | --- | --- |
| cel00030 | pathway | Pentose phosphate pathway - Caenorhabditis el... | 6.00 |
| cel00220 | pathway | Arginine biosynthesis - Caenorhabditis elegan... | 4.29 |
| cel00500 | pathway | Starch and sucrose metabolism - Caenorhabditi... | 3.99 |
| cel00330 | pathway | Arginine and proline metabolism - Caenorhabdi... | 2.29 |
| cel00020 | pathway | Citrate cycle (TCA cycle) - Caenorhabditis el... | 2.23 |
| cel00250 | pathway | Alanine, aspartate and glutamate metabolism -... | 2.01 |
| cel00480 | pathway | Glutathione metabolism - Caenorhabditis elega... | 1.84 |
| cel01200 | pathway | Carbon metabolism - Caenorhabditis elegans (n... | 1.74 |
| cel00534 | pathway | Glycosaminoglycan biosynthesis - heparan sulf... | 1.50 |
| M00006 | module | Pentose phosphate pathway, oxidative phase, g... | 6.00 |
| M00008 | module | Entner-Doudoroff pathway, glucose-6P => glyce... | 6.00 |
| M00012 | module | Glyoxylate cycle | 6.00 |
| M00059 | module | Glycosaminoglycan biosynthesis, heparan sulfa... | 6.00 |
| M00096 | module | C5 isoprenoid biosynthesis, non-mevalonate pa... | 6.00 |
| M00135 | module | GABA biosynthesis, eukaryotes, putrescine => ... | 6.00 |
| M00308 | module | Semi-phosphorylative Entner-Doudoroff pathway... | 6.00 |
| M00010 | module | Citrate cycle, first carbon oxidation, oxaloa... | 5.90 |
| M00004 | module | Pentose phosphate pathway (Pentose phosphate ... | 4.31 |
| M00134 | module | Polyamine biosynthesis, arginine => ornithine... | 4.05 |
| M00009 | module | Citrate cycle (TCA cycle, Krebs cycle) | 2.93 |
| M00173 | module | Reductive citrate cycle (Arnon-Buchanan cycle... | 2.88 |
| M00854 | module | Glycogen biosynthesis, glucose-1P => glycogen... | 2.60 |
| M00005 | module | PRPP biosynthesis, ribose 5P => PRPP | 2.49 |
| M00855 | module | Glycogen degradation, glycogen => glucose-6P | 1.56 |
| M00565 | module | Trehalose biosynthesis, D-glucose 1P => treha... | 1.51 |
| M00740 | module | Methylaspartate cycle | 1.48 |
| M00058 | module | Glycosaminoglycan biosynthesis, chondroitin s... | 1.36 |
| 1.1.1.21 | enzyme | aldose reductase | 6.00 |
| 1.1.1.343 | enzyme | phosphogluconate dehydrogenase (NAD+-dependen... | 6.00 |
| 1.1.1.44 | enzyme | phosphogluconate dehydrogenase (NADP+-depende... | 6.00 |
| 1.14.11.2 | enzyme | procollagen-proline 4-dioxygenase | 6.00 |
| 1.5.5.2 | enzyme | proline dehydrogenase | 6.00 |
| 2.3.3.9 | enzyme | malate synthase | 6.00 |
| 2.4.1.135 | enzyme | galactosylgalactosylxylosylprotein 3-beta-glu... | 6.00 |
| 2.4.1.225 | enzyme | N-acetylglucosaminyl-proteoglycan 4-beta-gluc... | 6.00 |
| 2.4.1.226 | enzyme | N-acetylgalactosaminyl-proteoglycan 3-beta-gl... | 6.00 |
| 2.7.1.12 | enzyme | gluconokinase | 6.00 |
| 3.1.1.31 | enzyme | 6-phosphogluconolactonase | 6.00 |
| 3.2.1.28 | enzyme | alpha,alpha-trehalase | 6.00 |
| 3.5.1.26 | enzyme | N4-(beta-N-acetylglucosaminyl)-L-asparaginase | 6.00 |
| 4.2.1.3 | enzyme | aconitate hydratase | 6.00 |
| 5.1.3.17 | enzyme | heparosan-N-sulfate-glucuronate 5-epimerase | 6.00 |
| 6.5.1.7 | enzyme | DNA ligase (ATP, ADP or GTP) | 6.00 |
| 4.1.3.1 | enzyme | isocitrate lyase | 5.94 |
| 1.7.1.7 | enzyme | GMP reductase | 4.62 |
| 2.3.3.1 | enzyme | citrate (Si)-synthase | 4.12 |
| 1.8.1.7 | enzyme | glutathione-disulfide reductase | 4.09 |
| 2.4.1.186 | enzyme | glycogenin glucosyltransferase | 3.52 |
| 2.4.1.224 | enzyme | glucuronosyl-N-acetylglucosaminyl-proteoglyca... | 3.36 |
| 2.4.1.223 | enzyme | glucuronosyl-galactosyl-proteoglycan 4-alpha-... | 3.36 |
| 4.1.1.17 | enzyme | ornithine decarboxylase | 3.32 |
| 1.1.1.49 | enzyme | glucose-6-phosphate dehydrogenase (NADP+) | 3.03 |
| 1.5.1.2 | enzyme | pyrroline-5-carboxylate reductase | 2.99 |
| 1.1.1.41 | enzyme | isocitrate dehydrogenase (NAD+) | 2.89 |
| 2.4.1.17 | enzyme | glucuronosyltransferase | 2.57 |
| 2.4.1.11 | enzyme | glycogen(starch) synthase | 2.35 |
| 1.11.1.27 | enzyme | glutathione-dependent peroxiredoxin | 2.24 |
| 6.3.5.4 | enzyme | asparagine synthase (glutamine-hydrolysing) | 2.21 |
| 1.1.1.22 | enzyme | UDP-glucose 6-dehydrogenase | 2.11 |
| 5.3.3.2 | enzyme | isopentenyl-diphosphate Delta-isomerase | 2.05 |
| 1.1.1.363 | enzyme | glucose-6-phosphate dehydrogenase [NAD(P)+] | 2.03 |
| 2.4.1.102 | enzyme | beta-1,3-galactosyl-O-glycosyl-glycoprotein b... | 1.97 |
| 2.7.7.9 | enzyme | UTP---glucose-1-phosphate uridylyltransferase | 1.87 |
| 2.6.1.45 | enzyme | serine---glyoxylate transaminase | 1.80 |
| 1.11.1.9 | enzyme | glutathione peroxidase | 1.80 |
| 6.3.5.2 | enzyme | GMP synthase (glutamine-hydrolysing) | 1.74 |
| 2.6.1.44 | enzyme | alanine---glyoxylate transaminase | 1.74 |
| 6.3.1.2 | enzyme | glutamine synthetase | 1.74 |
| 2.4.1.155 | enzyme | alpha-1,6-mannosyl-glycoprotein 6-beta-N-acet... | 1.73 |
| 6.4.1.1 | enzyme | pyruvate carboxylase | 1.72 |
| 2.4.1.141 | enzyme | N-acetylglucosaminyldiphosphodolichol N-acety... | 1.64 |
| 2.4.1.143 | enzyme | alpha-1,6-mannosyl-glycoprotein 2-beta-N-acet... | 1.58 |
| 2.4.1.122 | enzyme | N-acetylgalactosaminide beta-1,3-galactosyltr... | 1.57 |
| 4.1.1.35 | enzyme | UDP-glucuronate decarboxylase | 1.48 |
| 2.4.1.25 | enzyme | 4-alpha-glucanotransferase | 1.44 |
| 2.4.1.38 | enzyme | beta-N-acetylglucosaminylglycopeptide beta-1,... | 1.39 |
| 3.1.4.35 | enzyme | 3',5'-cyclic-GMP phosphodiesterase | 1.31 |
| 2.4.1.18 | enzyme | 1,4-alpha-glucan branching enzyme | 1.31 |

# **Table S3.** The differential gene expression analysis of nematodes in each group, Lobetyolin treated group vs. blank control.

|  | Gene Symbol | log_2_ (Lobetyolin / C) | -lg[Qvalue (Lobetyolin / C)] |  |  | Gene Symbol | log_2_ (Lobetyolin / C) | -lg[Qvalue (Lobetyolin / C)] |
| --- | --- | --- | --- | --- | --- | --- | --- | --- |
| 1 | *C37A5.3* | 1.78 | 2.73 |  | 163 | *nspc-16* | 2.29 | 50.62 |
| 2 | *F07E5.12* | −1.81 | 2.42 |  | 164 | *D1086.2* | 1.78 | 8.37 |
| 3 | *F19B10.13* | 1.61 | 10.89 |  | 165 | *oac-12* | −2.57 | 2.45 |
| 4 | *F52C12.6* | 4.57 | 3.28 |  | 166 | *E02H4.4* | 1.58 | 9.69 |
| 5 | *Y54G2A.57* | 2.33 | 4.58 |  | 167 | *EGAP2.2* | −1.81 | 3.90 |
| 6 | *K03B4.8* | 2.33 | 2.27 |  | 168 | *scl-14* | 2.01 | 2.71 |
| 7 | *Y60C6A.2* | 3.33 | 4.31 |  | 169 | *F10A3.1* | 2.72 | 6.87 |
| 8 | *ZC404.15* | 1.74 | 2.21 |  | 170 | *F12E12.6* | −2.09 | 2.39 |
| 9 | *F40D4.17* | −1.81 | 11.76 |  | 171 | *F14F9.2* | 4.51 | 6.66 |
| 10 | *F57G4.11* | 2.81 | 11.65 |  | 172 | *F14H3.5* | −1.67 | 21.27 |
| 11 | *C25F9.16* | 2.48 | 8.93 |  | 173 | *F18A12.7* | −1.55 | 7.25 |
| 12 | *B0416.10* | −2.9 | 22.71 |  | 174 | *F19B10.4* | 2.36 | 6.40 |
| 13 | *R09A8.9* | −1.59 | 5.41 |  | 175 | *F19C7.3* | −1.81 | 2.45 |
| 14 | *F20D1.22* | −3.08 | 106.21 |  | 176 | *nas-24* | 1.59 | 6.46 |
| 15 | *spp-17* | −1.98 | 92.58 |  | 177 | *F25D1.5* | −1.68 | 76.08 |
| 16 | *ceh-45* | 1.8 | 3.08 |  | 178 | *F26F2.1* | 1.53 | 3.60 |
| 17 | *ari-1.2* | −1.88 | 22.68 |  | 179 | *F26F2.3* | 2.81 | 3.56 |
| 18 | *acdh-1* | −3.64 | 6.23 |  | 180 | *col-152* | −2.12 | 10.50 |
| 19 | *C55B7.3* | −1.69 | 77.71 |  | 181 | *gst-38* | 2.11 | 22.32 |
| 20 | *col-60* | −1.54 | 84.30 |  | 182 | *fbxa-181* | −2.68 | 9.80 |
| 21 | *ercc-1* | −2.23 | 28.25 |  | 183 | *F35F10.5* | 1.63 | 4.12 |
| 22 | *Y106G6D.2* | −1.85 | 4.37 |  | 184 | *gst-24* | 1.63 | 25.15 |
| 23 | *vet-2* | −1.78 | 9.04 |  | 185 | *nspb-2* | −1.57 | 2.18 |
| 24 | *T02G6.5* | −2.04 | 19.61 |  | 186 | *nep-15* | −2.32 | 81.33 |
| 25 | *Y47H9C.1* | −1.59 | 16.34 |  | 187 | *grl-27* | −1.54 | 2.79 |
| 26 | *C47F8.1* | −2.05 | 7.49 |  | 188 | *nspc-13* | 1.67 | 22.41 |
| 27 | *C54C8.4* | −1.95 | 3.19 |  | 189 | *F44G3.10* | 1.53 | 2.68 |
| 28 | *clec-15* | 3.36 | 157.94 |  | 190 | *btb-7* | −1.81 | 8.22 |
| 29 | *clec-13* | 3.36 | 157.94 |  | 191 | *pals-31* | 1.58 | 9.38 |
| 30 | *vet-6* | −1.84 | 6.27 |  | 192 | *scl-11* | 5.16 | 2.84 |
| 31 | *sepa-1* | −2.59 | 17.90 |  | 193 | *clec-240* | −5.26 | 2.87 |
| 32 | *T04D3.1* | −1.66 | 19.92 |  | 194 | *col-133* | −1.89 | 88.43 |
| 33 | *sdz-30* | −2.33 | 5.50 |  | 195 | *fbxb-103* | −2.04 | 4.00 |
| 34 | *Y71A12B.11* | −1.51 | 4.18 |  | 196 | *F53F1.3* | 3.08 | 4.26 |
| 35 | *fbxc-32* | −1.69 | 11.83 |  | 197 | *dot-1.2* | 2.49 | 2.42 |
| 36 | *sdz-9* | −2.13 | 2.68 |  | 198 | *col-156* | −1.92 | 55.17 |
| 37 | *T06D4.1* | −1.51 | 44.95 |  | 199 | *F57B9.3* | 2.17 | 12.05 |
| 38 | *C46E10.8* | −2.2 | 14.18 |  | 200 | *F58E1.13* | −1.81 | 6.31 |
| 39 | *T24E12.5* | 1.58 | 43.76 |  | 201 | *clec-206* | −2.44 | 5.86 |
| 40 | *T24E12.11* | −1.92 | 5.29 |  | 202 | *H17B01.2* | −3.36 | 19.54 |
| 41 | *slc-36.5* | 1.65 | 32.51 |  | 203 | *parg-2* | 2.48 | 83.51 |
| 42 | *E04F6.9* | 1.55 | 48.14 |  | 204 | *H37A05.2* | −1.86 | 7.02 |
| 43 | *arrd-2* | −1.59 | 3.52 |  | 205 | *K01D12.9* | −1.55 | 11.42 |
| 44 | *pap-2* | −2.99 | 6.09 |  | 206 | *K02E2.7* | 1.62 | 4.59 |
| 45 | *chil-8* | 5.86 | 4.18 |  | 207 | *K04C1.5* | −1.67 | 53.64 |
| 46 | *clec-61* | 2.7 | 129.51 |  | 208 | *K05F6.10* | 2.04 | 9.75 |
| 47 | *F54D5.4* | −1.52 | 4.76 |  | 209 | *cyp-13B2* | −1.51 | 5.16 |
| 48 | *pap-3* | −1.96 | 4.48 |  | 210 | *K08D8.1* | 1.68 | 2.88 |
| 49 | *tag-276* | −1.58 | 2.25 |  | 211 | *K09E3.7* | −1.74 | 11.72 |
| 50 | *pes-10* | −1.72 | 2.12 |  | 212 | *M60.4* | 1.56 | 55.84 |
| 51 | *Y48E1B.8* | −1.72 | 3.97 |  | 213 | *M151.2* | −2.34 | 11.80 |
| 52 | *F01D5.7* | 1.87 | 14.34 |  | 214 | *M151.3* | −1.77 | 8.99 |
| 53 | *Y48B6A.10* | −1.58 | 7.33 |  | 215 | *ztf-14* | 1.81 | 4.82 |
| 54 | *col-88* | −1.87 | 100.58 |  | 216 | *R05C11.2* | 1.98 | 5.28 |
| 55 | *gsto-3* | 1.73 | 10.72 |  | 217 | *math-38* | 1.95 | 50.06 |
| 56 | *C18F10.2* | 1.56 | 3.51 |  | 218 | *T09B4.6* | 1.59 | 6.68 |
| 57 | *numr-1* | 1.82 | 33.24 |  | 219 | *T10C6.7* | −1.54 | 58.54 |
| 58 | *numr-2* | 1.82 | 33.24 |  | 220 | *lips-12* | −2.16 | 2.51 |
| 59 | *tbx-9* | −1.56 | 11.75 |  | 221 | *lgc-54* | 1.62 | 5.45 |
| 60 | *dsl-2* | −1.87 | 8.71 |  | 222 | *T16G1.3* | −2.4 | 17.17 |
| 61 | *ilys-3* | 2.67 | 55.18 |  | 223 | *zig-13* | 2.43 | 5.37 |
| 62 | *clec-82* | 2.06 | 55.12 |  | 224 | *ugt-10* | 2.28 | 4.75 |
| 63 | *gba-4* | −2.4 | 161.15 |  | 225 | *T20D4.7* | 2.11 | 15.88 |
| 64 | *C06G3.3* | 2.33 | 12.09 |  | 226 | *pgp-7* | 2.12 | 5.79 |
| 65 | *mes-6* | 6.53 | 5.58 |  | 227 | *T22B2.1* | −1.67 | 4.50 |
| 66 | *col-120* | −1.81 | 120.58 |  | 228 | *str-124* | 1.7 | 5.83 |
| 67 | *F56D5.5* | −2.57 | 8.00 |  | 229 | *nas-27* | −2.04 | 5.06 |
| 68 | *hsp-12.3* | 2.5 | 25.65 |  | 230 | *T24A6.7* | 2.7 | 29.41 |
| 69 | *gadr-2* | −2.26 | 6.11 |  | 231 | *enri-2* | −1.78 | 6.60 |
| 70 | *col-126* | −2.16 | 18.39 |  | 232 | *T26H5.8* | 2.28 | 36.43 |
| 71 | *cbd-1* | −1.54 | 128.04 |  | 233 | *T27A8.2* | −1.61 | 5.16 |
| 72 | *scl-2* | 1.91 | 222.31 |  | 234 | *pals-29* | 3.65 | 2.01 |
| 73 | *JC8.4* | −1.75 | 10.45 |  | 235 | *T28A11.19* | 2.18 | 14.81 |
| 74 | *ccch-5* | −1.7 | 3.86 |  | 236 | *W01C9.2* | 2.19 | 20.94 |
| 75 | *clec-190* | −3.27 | 19.93 |  | 237 | *W04A4.2* | −1.86 | 98.45 |
| 76 | *Y105C5A.13* | 1.68 | 8.36 |  | 238 | *txt-3* | 3.01 | 7.48 |
| 77 | *Y116A8C.19* | −3.07 | 2.27 |  | 239 | *flh-3* | −1.62 | 27.50 |
| 78 | *skr-9* | −2.14 | 4.84 |  | 240 | *fbxa-211* | −1.72 | 84.71 |
| 79 | *skr-8* | −1.67 | 4.17 |  | 241 | *clec-4* | −1.64 | 40.72 |
| 80 | *gln-5* | −1.52 | 68.31 |  | 242 | *Y39A1A.16* | −2.41 | 3.30 |
| 81 | *W07B8.4* | 1.77 | 12.50 |  | 243 | *Y39A3A.2* | 2.97 | 2.73 |
| 82 | *F22F7.7* | 1.64 | 58.74 |  | 244 | *Y39F10A.3* | 1.64 | 5.30 |
| 83 | *C45H4.14* | −2.3 | 4.81 |  | 245 | *srd-23* | 2.07 | 2.18 |
| 84 | *Y73C8C.8* | −1.85 | 6.76 |  | 246 | *nlp-25* | −1.97 | 13.97 |
| 85 | *ceh-49* | −1.69 | 12.19 |  | 247 | *dmd-3* | 1.76 | 5.18 |
| 86 | *W06H8.2* | 1.83 | 32.30 |  | 248 | *Y44A6B.3* | −1.68 | 6.26 |
| 87 | *F20A1.4* | 1.54 | 2.87 |  | 249 | *clec-76* | 2.39 | 48.41 |
| 88 | *ftn-1* | 2.72 | 16.29 |  | 250 | *Y47H10A.2* | −1.83 | 4.71 |
| 89 | *oma-2* | −1.67 | 105.78 |  | 251 | *Y51H4A.8* | 1.53 | 12.43 |
| 90 | *W02D7.11* | 2.47 | 4.97 |  | 252 | *col-137* | −3.72 | 42.01 |
| 91 | *B0507.8* | 2.76 | 25.13 |  | 253 | *scl-17* | 2.71 | 3.31 |
| 92 | *hrg-3* | 1.8 | 13.77 |  | 254 | *Y58A7A.5* | 2.85 | 184.68 |
| 93 | *C45B11.2* | 1.59 | 10.07 |  | 255 | *thn-5* | 2.56 | 26.09 |
| 94 | *clec-222* | −1.62 | 15.10 |  | 256 | *Y60A9.3* | −2.95 | 3.93 |
| 95 | *clec-223* | −1.64 | 38.98 |  | 257 | *Y62H9A.11* | 1.67 | 2.73 |
| 96 | *C44H9.7* | 1.82 | 3.92 |  | 258 | *oac-56* | 1.97 | 8.69 |
| 97 | *col-161* | −1.66 | 100.36 |  | 259 | *Y69A2AR.12* | 1.84 | 4.89 |
| 98 | *col-162* | −1.76 | 96.46 |  | 260 | *Y69A2AR.25* | 2.03 | 6.64 |
| 99 | *bath-39* | 3.27 | 2.34 |  | 261 | *clec-9* | 1.7 | 6.31 |
| 100 | *C06B3.7* | 1.84 | 33.05 |  | 262 | *clec-210* | −1.55 | 11.38 |
| 101 | *cest-1.1* | 1.53 | 35.06 |  | 263 | *Y73C8C.3* | −1.85 | 4.45 |
| 102 | *F14D7.2* | −1.57 | 40.34 |  | 264 | *Y73C8C.4* | 1.96 | 3.55 |
| 103 | *hil-1* | 1.73 | 23.92 |  | 265 | *Y82E9BL.3* | 4.55 | 44.72 |
| 104 | *clec-47* | 2.64 | 215.86 |  | 266 | *Y105C5B.3* | 1.61 | 26.85 |
| 105 | *dhs-23* | −2.11 | 123.92 |  | 267 | *Y106G6H.9* | −1.78 | 11.09 |
| 106 | *F14H3.3* | −1.71 | 16.41 |  | 268 | *fbxa-75* | 3.15 | 2.03 |
| 107 | *F14H3.4* | −1.5 | 23.65 |  | 269 | *droe-8* | 2.14 | 7.15 |
| 108 | *F11A5.9* | 1.71 | 116.28 |  | 270 | *clec-60* | 3.33 | 273.45 |
| 109 | *cyp-37B1* | −1.62 | 75.66 |  | 271 | *clec-143* | 1.54 | 8.09 |
| 110 | *txt-14* | 1.57 | 7.82 |  | 272 | *ZK899.5* | −2.67 | 2.37 |
| 111 | *ins-35* | 2.31 | 13.73 |  | 273 | *ZK899.6* | −1.98 | 3.12 |
| 112 | *Y75D11A.3* | −1.54 | 10.26 |  | 274 | *gpa-9* | 1.58 | 2.64 |
| 113 | *rgs-8.1* | −1.68 | 11.44 |  | 275 | *hen-1* | 1.81 | 3.60 |
| 114 | *meg-3* | −1.51 | 46.82 |  | 276 | *sri-40* | −1.52 | 24.86 |
| 115 | *Y40A1A.3* | −1.56 | 5.23 |  | 277 | *tsp-17* | 1.77 | 2.25 |
| 116 | *lbp-1* | −11.02 | 18.55 |  | 278 | *twk-4* | 1.68 | 4.20 |
| 117 | *amt-1* | 1.69 | 19.16 |  | 279 | *zig-3* | 1.98 | 19.65 |
| 118 | *zig-4* | 1.91 | 52.25 |  | 280 | *C23H5.15* | 1.5 | 20.31 |
| 119 | *asns-2* | 1.65 | 113.52 |  | 281 | *C42D4.19* | 2.6 | 17.00 |
| 120 | *R07A4.3* | 2.06 | 5.37 |  | 282 | *K04A8.20* | 2.42 | 4.59 |
| 121 | *pgp-8* | 2.25 | 42.81 |  | 283 | *C33A12.19* | 2.41 | 44.68 |
| 122 | *pgp-6* | 2.1 | 237.13 |  | 284 | *F18E3.11* | 2.03 | 10.26 |
| 123 | *nspc-12* | 1.66 | 37.49 |  | 285 | *D1086.12* | 1.56 | 38.88 |
| 124 | *nspc-14* | 1.61 | 37.57 |  | 286 | *nspc-18* | 2.04 | 22.53 |
| 125 | *nspc-15* | 1.55 | 24.90 |  | 287 | *Y54G2A.37* | 2.47 | 8.03 |
| 126 | *R03G8.6* | −1.62 | 27.97 |  | 288 | *gst-34* | 2.16 | 4.69 |
| 127 | *R04D3.3* | −1.62 | 60.86 |  | 289 | *ddn-1* | 2.39 | 49.06 |
| 128 | *mxl-3* | 1.53 | 74.85 |  | 290 | *F11A5.13* | 2.14 | 2.71 |
| 129 | *fkh-3* | −1.68 | 7.94 |  | 291 | *asp-16* | 2.08 | 7.57 |
| 130 | *fkh-4* | −1.61 | 5.14 |  | 292 | *Y47D7A.15* | −2.22 | 151.22 |
| 131 | *F23D12.2* | −1.93 | 11.76 |  | 293 | *spsb-2* | −1.5 | 79.30 |
| 132 | *nspc-17* | 2.05 | 33.77 |  | 294 | *C49G7.7* | 1.7 | 10.82 |
| 133 | *nspc-20* | 1.59 | 63.46 |  | 295 | *Y46C8AM.1* | 1.64 | 23.71 |
| 134 | *hch-1* | −4.11 | 2.54 |  | 296 | *Y60C6A.3* | 3.08 | 3.53 |
| 135 | *rgs-11* | −1.5 | 13.91 |  | 297 | *T26H5.9* | 1.68 | 90.84 |
| 136 | *B0281.4* | −2.18 | 12.46 |  | 298 | *Y58A7A.4* | 2.57 | 118.10 |
| 137 | *pals-28* | 3.17 | 2.52 |  | 299 | *C30H6.12* | 2.13 | 2.04 |
| 138 | *B0348.2* | 1.67 | 2.70 |  | 300 | *F40H7.12* | 2.25 | 2.63 |
| 139 | *clec-2* | 3.21 | 3.85 |  | 301 | *col-135* | −1.67 | 34.52 |
| 140 | *B0507.7* | 2.69 | 9.53 |  | 302 | *gpd-2* | 3.55 | 9.38 |
| 141 | *C07G1.7* | −1.7 | 21.33 |  | 303 | *nspc-19* | 1.9 | 26.94 |
| 142 | *irg-1* | 2.12 | 62.83 |  | 304 | *C04A11.5* | 2.64 | 9.45 |
| 143 | *fbxa-163* | 2.48 | 62.34 |  | 305 | *F14D7.10* | 1.84 | 6.78 |
| 144 | *fbxa-164* | 3.59 | 26.41 |  | 306 | *ZC412.3* | 1.6 | 64.95 |
| 145 | *fbxa-165* | 2.64 | 12.58 |  | 307 | *Y17G7B.23* | 1.83 | 3.12 |
| 146 | *C08E8.4* | 2.17 | 55.22 |  | 308 | *Y45F10D.14* | −1.86 | 5.00 |
| 147 | *C08F11.3* | 1.76 | 18.52 |  | 309 | *pudl-2* | 2.42 | 2.56 |
| 148 | *fbxc-58* | 1.66 | 47.78 |  | 310 | *lgc-29* | −2.46 | 2.99 |
| 149 | *gpx-3* | 1.61 | 14.15 |  | 311 | *T07H8.11* | 2.36 | 18.70 |
| 150 | *C11G6.2* | 2.04 | 5.19 |  | 312 | *F41G4.13* | 1.53 | 2.33 |
| 151 | *gsto-1* | 1.66 | 41.00 |  | 313 | *B0252.11* | 2.07 | 2.73 |
| 152 | *C32H11.9* | −2.38 | 7.37 |  | 314 | *C16C8.17* | 2.95 | 2.67 |
| 153 | *dod-21* | −2.26 | 9.93 |  | 315 | *T26H5.10* | 1.84 | 13.80 |
| 154 | *clec-6* | 2.01 | 9.79 |  | 316 | *B0563.9* | 2.54 | 23.22 |
| 155 | *fipr-24* | 2.07 | 10.11 |  | 317 | *F13E9.15* | 1.93 | 47.04 |
| 156 | *clec-3* | 3.33 | 37.70 |  | 318 | *C25F9.11* | 2.07 | 51.59 |
| 157 | *C44B12.3* | −1.6 | 3.83 |  | 319 | *C49G7.12* | 1.91 | 33.75 |
| 158 | *srbc-20* | 1.88 | 4.17 |  | 320 | *F18E3.12* | 2.72 | 3.38 |
| 159 | *irg-2* | 1.64 | 8.48 |  | 321 | *F20E11.17* | 6.26 | 14.83 |
| 160 | *C52A10.3* | −2.25 | 5.40 |  | 322 | *R13H4.2* | 1.57 | 106.39 |
| 161 | *C53A5.9* | 1.91 | 2.20 |  | 323 | *T10C6.16* | −1.99 | 2.60 |
| 162 | *lys-9* | 3.91 | 7.65 |  |  |  |  |  |

*Entries highlighted with a yellow background indicate key differentially expressed genes (DEGs).

**Table S4.** The enriched pathways of DEGs upon Lobetyolin treatment analyzed by KEGG pathway enrichment analysis.

|  | KEGG Pathway Term Desc | Rich Ratio | -lg(*p* value) | Gene Symbols |
| --- | --- | --- | --- | --- |
| 1 | Glutathione metabolism | 0.088 | 4.67 | *F22F7.7， gpx-3， gsto-1， gst-38 gst-24* |
| 2 | Platinum drug resistance | 0.078 | 3.60 | *ercc-1， gsto-1， gst-38， gst-24* |
| 3 | Pathways in cancer | 0.032 | 3.12 | *skr-9， skr-8， mxl-3， gsto-1， gst-38， gst-24* |
| 4 | Chemical carcinogenesis - DNA adducts | 0.070 | 2.63 | *gsto-1， gst-38， gst-24* |
| 5 | Metabolism of xenobiotics by cytochrome P450 | 0.064 | 2.52 | *gsto-1， gst-38， gst-24* |
| 6 | Drug metabolism - cytochrome P450 | 0.058 | 2.39 | *gsto-1， gst-38， gst-24* |
| 7 | Drug metabolism - other enzymes | 0.047 | 2.14 | *gsto-1， gst-38， gst-24* |
| 8 | Biosynthesis of amino acids | 0.042 | 2.01 | *gln-5， asns-2， gpd-2* |
| 9 | Chemical carcinogenesis - receptor activation | 0.042 | 2.01 | *gsto-1， gst-38， gst-24* |
| 10 | Fluid shear stress and atherosclerosis | 0.037 | 1.84 | *gsto-1， gst-38， gst-24* |
| 11 | Alanine, aspartate and glutamate metabolism | 0.065 | 1.79 | *gln-5， asns-2* |
| 12 | Circadian rhythm | 0.065 | 1.79 | *skr-9， skr-8* |
| 13 | Hepatocellular carcinoma | 0.034 | 1.77 | *gsto-1， gst-38， gst-24* |
| 14 | Longevity regulating pathway - worm | 0.033 | 1.73 | *gsto-1， gst-38， gst-24* |
| 15 | Hedgehog signaling pathway - fly | 0.049 | 1.56 | *skr-9， skr-8* |
| 16 | TGF-beta signaling pathway | 0.047 | 1.52 | *skr-9， skr-8* |
| 17 | Necroptosis | 0.032 | 1.23 | *gln-5， ftn-1* |
| 18 | Chemical carcinogenesis - reactive oxygen species | 0.021 | 1.20 | *gsto-1， gst-38， gst-24* |
| 19 | Transcriptional misregulation in cancer | 0.031 | 1.20 | *mxl-3， dot-1.2* |
| 20 | Salmonella infection | 0.020 | 1.15 | *skr-9， skr-8， gpd-2* |
| 21 | Nitrogen metabolism | 0.083 | 1.13 | *gln-5* |
| 22 | Wnt signaling pathway | 0.026 | 1.06 | *skr-9， skr-8* |
| 23 | mRNA surveillance pathway | 0.025 | 1.05 | *pap-2， pap-3* |
| 24 | Arginine biosynthesis | 0.059 | 0.98 | *gln-5* |
| 25 | Other glycan degradation | 0.059 | 0.98 | *gba-4* |
| 26 | Oocyte meiosis | 0.022 | 0.94 | *skr-9， skr-8* |
| 27 | Arachidonic acid metabolism | 0.053 | 0.94 | *gpx-3* |
| 28 | Ubiquitin mediated proteolysis | 0.022 | 0.94 | *skr-9， skr-8* |
| 29 | Cell cycle | 0.022 | 0.93 | *skr-9， skr-8* |
| 30 | Ferroptosis | 0.042 | 0.84 | *ftn-1* |
| 31 | Small cell lung cancer | 0.042 | 0.84 | *mxl-3* |
| 32 | Human immunodeficiency virus 1 infection | 0.019 | 0.84 | *skr-9， skr-8* |
| 33 | Lysosome | 0.019 | 0.83 | *gba-4， W07B8.4* |
| 34 | Fanconi anemia pathway | 0.040 | 0.83 | *ercc-1* |
| 35 | ABC transporters | 0.033 | 0.75 | *pgp-6* |
| 36 | Mineral absorption | 0.032 | 0.74 | *ftn-1* |
| 37 | Antigen processing and presentation | 0.031 | 0.73 | *W07B8.4* |
| 38 | Thyroid hormone synthesis | 0.031 | 0.73 | *gpx-3* |
| 39 | Glyoxylate and dicarboxylate metabolism | 0.028 | 0.68 | *gln-5* |
| 40 | Nucleotide excision repair | 0.027 | 0.67 | *ercc-1* |
| 41 | Protein processing in endoplasmic reticulum | 0.015 | 0.66 | *skr-9， skr-8* |
| 42 | Sphingolipid metabolism | 0.026 | 0.65 | *gba-4* |
| 43 | Renin secretion | 0.026 | 0.65 | *W07B8.4* |
| 44 | Bile secretion | 0.026 | 0.65 | *pgp-6* |
| 45 | Lysine degradation | 0.025 | 0.64 | *dot-1.2* |
| 46 | GABAergic synapse | 0.024 | 0.63 | *gln-5* |
| 47 | Glycolysis / Gluconeogenesis | 0.024 | 0.62 | *gpd-2* |
| 48 | NOD-like receptor signaling pathway | 0.024 | 0.62 | *W07B8.4* |
| 49 | Glutamatergic synapse | 0.023 | 0.61 | *gln-5* |
| 50 | Shigellosis | 0.013 | 0.60 | *skr-9， skr-8* |
| 51 | HIF-1 signaling pathway | 0.020 | 0.57 | *gpd-2* |
| 52 | Fatty acid degradation | 0.019 | 0.54 | *acdh-1* |
| 53 | Valine, leucine and isoleucine degradation | 0.019 | 0.54 | *acdh-1* |
| 54 | Gastric cancer | 0.019 | 0.54 | *pgp-6* |
| 55 | Apoptosis | 0.017 | 0.50 | *W07B8.4* |
| 56 | MicroRNAs in cancer | 0.016 | 0.49 | *pgp-6* |
| 57 | Fatty acid metabolism | 0.015 | 0.45 | *acdh-1* |
| 58 | MAPK signaling pathway | 0.013 | 0.39 | *mxl-3* |
| 59 | Pathogenic Escherichia coli infection | 0.012 | 0.38 | *gpd-2* |
| 60 | Autophagy - animal | 0.011 | 0.35 | *W07B8.4* |
| 61 | Carbon metabolism | 0.0093 | 0.30 | *gpd-2* |
| 62 | Diabetic cardiomyopathy | 0.0074 | 0.23 | *gpd-2* |
| 63 | Huntington disease | 0.0048 | 0.12 | *gpx-3* |
| 64 | Amyotrophic lateral sclerosis | 0.0042 | 0.10 | *gpx-3* |
| 65 | Alzheimer disease | 0.0041 | 0.10 | *gpd-2* |
| 66 | Pathways of neurodegeneration - multiple diseases | 0.0036 | 0.072 | *gpx-3* |

*Entries highlighted with a yellow background indicate key differentially expressed genes (DEGs).

**Figure S2.** The calibration curve for Lobetyolin.

**Table S5.** The content of Lobetyolin in different Chinese medicinal herbs.

| Latin name | Part | Average sample content (mg/100g plant) |
| --- | --- | --- |
| *Codonopsis pilosula (Franch.)Nannf.* | root | 92.2 ± 0.6 |
| *Codonopsis tangshen Oliv.* | root | 21.9 ± 0.06 |
| *Codonopsis pilosula Nannf.var.modesta（Nannf.）L.T.Shen* | root | 135.9 ± 0.1 |
| *Adenophora tetraphylla(Thunb.)Fisch.* | root | 6.0 ± 0.4 |
| *Cyclocodon lancifolius* | fruit | 132.3 ± 0.2 |

**Figure S3.** 1H, 13C NMR and HR ESI MS of Lobetyolin.

1. ^1^H NMR

1. ^13^C NMR

**Table S6.** The strains and culture temperature of *C. elegans.*

| *C. elegans* strains | Genotype | Culture temperature（℃） |
| --- | --- | --- |
| N2 | wild-type | 20 |
| CL4176 | dvIs27 [*myo-3p::*Aβ_1-42_*::let-851 3'UTR） + rol-6（su1006）*] | 15 |
| CL2006 | dvIs2 [*pCL12（unc-54/*human Aβ_1-42_ minigene*） + rol-6（su1006）*] | 20 |
| CL2122 | dvIs15 [*（pPD30.38） unc-54（vector） + （pCL26） mtl-2::GFP*] | 15 |
| CL2355 | dvIs50 [*pCL45 （snb-1::*Aβ_1-42_*::3' UTR（long） + mtl-2::GFP*] | 15 |
